# Increased bundle‐sheath leakiness of CO_2_ during photosynthetic induction shows a lack of coordination between the C_4_ and C_3_ cycles

**DOI:** 10.1111/nph.18485

**Published:** 2022-09-30

**Authors:** Yu Wang, Samantha S. Stutz, Carl J. Bernacchi, Ryan A. Boyd, Donald R. Ort, Stephen P. Long

**Affiliations:** ^1^ The Carl R. Woese Institute for Genomic Biology University of Illinois Urbana‐Champaign 1206 W Gregory Dr Urbana IL 61801 USA; ^2^ DOE Center for Advanced Bioenergy and Bioproducts Innovation University of Illinois at Urbana‐Champaign Urbana IL 61801 USA; ^3^ USDA‐ARS Global Change and Photosynthesis Research Unit University of Illinois at Urbana‐Champaign Urbana IL 61801 USA; ^4^ Departments of Plant Biology and Crop Sciences University of Illinois at Urbana‐Champaign Urbana IL 61801 USA; ^5^ Lancaster Environment Centre Lancaster University Lancaster LA1 4YQ UK

**Keywords:** bundle‐sheath leakage, C_4_ photosynthesis, carbon isotope discrimination, maize, photosynthetic efficiency, photosynthetic induction, sorghum, tunable diode laser absorption spectroscopy

## Abstract

- Use of a complete dynamic model of NADP‐malic enzyme C_4_ photosynthesis indicated that, during transitions from dark or shade to high light, induction of the C_4_ pathway was more rapid than that of C_3_, resulting in a predicted transient increase in bundle‐sheath CO_2_ leakiness (*ϕ*).
- Previously, *ϕ* has been measured at steady state; here we developed a new method, coupling a tunable diode laser absorption spectroscope with a gas‐exchange system to track *ϕ* in sorghum and maize through the nonsteady‐state condition of photosynthetic induction.
- In both species, *ϕ* showed a transient increase to > 0.35 before declining to a steady state of 0.2 by 1500 s after illumination. Average *ϕ* was 60% higher than at steady state over the first 600 s of induction and 30% higher over the first 1500 s.
- The transient increase in *ϕ*, which was consistent with model prediction, indicated that capacity to assimilate CO_2_ into the C_3_ cycle in the bundle sheath failed to keep pace with the rate of dicarboxylate delivery by the C_4_ cycle. Because nonsteady‐state light conditions are the norm in field canopies, the results suggest that *ϕ* in these major crops in the field is significantly higher and energy conversion efficiency lower than previous measured values under steady‐state conditions.

## Introduction

Photosynthetic energy conversion efficiency (*ε*_c_), the efficiency with which crops convert intercepted radiation into biomass, is a major limitation to the yield potential for both C_3_ and C_4_ crops (Zhu *et al*., 2008, 2010; Long *et al*., 2015). The *ε*_c_ of C_4_ species has the intrinsic advantage of minimizing energy loss to photorespiration under most conditions, compared with C_3_ species (Long & Spence, 2013). Although only 3% of species use the C_4_ pathway, they account for 23% of terrestrial gross primary productivity (Sage *et al*., 2012). C_4_ species are also overrepresented in agricultural production in which just three C_4_ crops (maize, sugarcane and sorghum) account for 32% of global production (Long & Spence, 2013; FAO *et al*., 2020). All three are from a single C_4_ evolutionary clade, tribe Andropogoneae, and use the NADP malic enzyme (ME) for decarboxylation in the bundle sheath. Despite high productivity, even under optimum conditions, these C_4_ crops still fall well short of the theoretical maximum energy conversion efficiency of 6% in the field (Zhu *et al*., 2008, 2010; Dohleman & Long, 2009). Understanding the limitations to realizing the theoretical maximum in field conditions is key to increasing the productivity of C_4_ crops.

C_4_ photosynthesis includes a light energy‐driven CO_2_‐concentrating mechanism that increases the CO_2_ concentration around Ribulose‐1,5‐bisphosphate carboxylase/oxygenase (Rubisco) in bundle‐sheath cells, competitively inhibiting the oxygenation reaction, with the result that photorespiration is almost eliminated under normal conditions (Hatch, 1978, 1987; Edwards & Walker, 1983; Keeley & Rundel, 2003; Sage, 2004). Compared with C_3_ photosynthesis, C_4_ photosynthesis requires two additional ATP per CO_2_ assimilated in the regeneration of phosphoenolpyruvate (PEP), the initial acceptor molecule for CO_2_ in the mesophyll. However, as the bundle sheath is not hermetically sealed, an inevitable consequence of the high [CO_2_] gradient formed between bundle sheath and mesophyll cells is leakiness (*ϕ*). Leakiness describes the proportion of carbon fixed by PEP carboxylase (PEPC) and released by decarboxylation in the bundle sheath that diffuses back to the mesophyll. A variety of methods have estimated an average *ϕ* of 0.2 in C_4_ NADP‐ME species when measured at steady state in high light (Kromdijk *et al*., 2014). This means that for every five CO_2_ molecules released by decarboxylation of malate in the bundle sheath, one will diffuse back to the mesophyll, raising the cost per net CO_2_ assimilated by 0.5 ATP. Minimizing *ϕ* requires close coordination between the C_3_ and C_4_ cycles. Any elevation of *ϕ* indicates some lack of coordination between the two photosynthetic cycles and therefore a loss of photosynthetic efficiency (Henderson *et al*., 1992).

Although previous studies of C_4_ leakiness have focused on steady‐state conditions (Bellasio & Griffiths, 2014; Kromdijk *et al*., 2014; von Caemmerer & Furbank, 2016), leaves in crop fields are seldom under steady‐state conditions; instead these crop species experience frequent fluctuations in environmental conditions, especially light intensity. Intermittent cloud cover, the movement of leaves, and the changing solar angle over the course of a day cause dramatic and often abrupt changes in the light environment, including sunflecking within the crop canopy (Pearcy, 1990; Zhu *et al*., 2004; Slattery *et al*., 2018; Ohkubo *et al*., 2020; Sakoda *et al*., 2020; Wang *et al*., 2020; Qiao *et al*., 2021; Long *et al*., 2022). The planting densities of these crops are increasing such that self‐shading and more frequent light fluctuations will continue to increase. Although light fluctuations at points on a leaf can occur in fractions of a second, the photosynthetic apparatus may require many minutes to adjust, potentially leading to losses of efficiency at the crop canopy level. This has led to a growing awareness of the need to address photosynthetic efficiency in fluctuating light (Hubbart *et al*., 2012; McAusland *et al*., 2016; Deans *et al*., 2019; Acevedo‐Siaca *et al*., 2020; De Souza *et al*., 2020; McAusland & Murchie, 2020; Murchie & Ruban, 2020). Much progress has been made in understanding the dynamic response to light in C_3_ plants in the past few years. Photosynthetic induction of C_3_ plants during shade‐to‐sun transitions is mainly influenced by three factors: activation of Rubisco, the speed of stomatal opening, and activation of the enzymes involved in RuBP regeneration within the C_3_ cycle (Pearcy, 1994; Mott & Woodrow, 2000; Kaiser *et al*., 2016; Slattery *et al*., 2018; Taylor *et al*., 2022). Photosynthetic rate during induction is lower than that under steady‐state; however, the major factors limiting photosynthesis, primarily Rubisco activation and stomatal opening, vary among crop species and all represent a loss of potential efficiency (McAusland *et al*., 2016; Taylor & Long, 2017; Acevedo‐Siaca *et al*., 2020, 2021; De Souza *et al*., 2020).

When grown under fluctuating light, two C_4_ species (*Setaria macrostachya* and *Amaranthus caudatus*) showed a greater reduction in biomass than that observed in two C_3_ species (*Triticum aestivum* and *Celosia argentea*) relative to growth under steady‐state light (Kubásek *et al*., 2013). As rapid stomatal movement was reported in C_4_ plants (Bellasio *et al*., 2017; Ozeki *et al*., 2022), the biomass reduction suggests that C_4_ species may be more vulnerable to efficiency losses under fluctuating light, perhaps because of the need to coordinate between the two photosynthetic cycles. This finding was challenged by Lee *et al*. (2022) who compared carbon assimilation during fluctuating light to steady‐state across six C_3_ and six C_4_ species. Whereas Kubásek *et al*. (2013) made measurements during photosynthetic induction, Lee *et al*. (2022) examined plants that were fully acclimated to high light and suggested that differences between the two studies could be a result of photosynthetic induction causing lower coordination between C_3_ and C_4_ cycles.

A dynamic modeling simulation of C_4_ photosynthetic induction coupled with gas‐exchange measurements identified Rubisco activase, PPDK regulatory protein and stomatal conductance as the major limitations to the efficiency of NADP‐ME‐type photosynthesis during dark to high‐light fluctuations. The degree of influence of these limiting factors varied somewhat among single accessions of maize, sorghum and sugarcane (Wang *et al*., 2021). Owing to the complex compartmentation of the photosynthetic reactions between mesophyll and bundle‐sheath cells, the gas‐exchange measurements in Wang *et al*. (2021) were not able directly to investigate the relationship between the C_4_ and C_3_ cycles or determine leakiness during induction. However, a higher leakiness was predicted during induction compared with the steady state, as activation of the C_4_ dicarboxylate cycle appeared significantly faster than that of Rubisco in the bundle sheath, based on available kinetic data (Wang *et al*., 2021).

Steady‐state *ϕ* increases only slightly with decreasing light and varies little when measured at different [CO_2_], suggesting the C_3_ and C_4_ cycles are well coordinated under steady‐state conditions (Henderson *et al*., 1992; Ubierna *et al*., 2011, 2013; Bellasio & Griffiths, 2014; Kromdijk *et al*., 2014). However, little is known about how *ϕ* changes under nonsteady‐state conditions. Leakiness can be estimated by including measurements of photosynthetic carbon isotope discrimination (Kromdijk *et al*., 2014). Estimates of leakiness using stable isotopes compare the theoretical model of photosynthetic discrimination (Δ^13^C) (Farquhar, 1983; Farquhar & Cernusak, 2012) with measured photosynthetic discrimination (Δ^13^C_obs_) (Kromdijk *et al*., 2014). Stable isotope discrimination can be estimated in real‐time using a tunable diode laser absorption spectroscope (TDL) coupled to a gas‐exchange system (Barbour *et al*., 2007). In steady‐state measurements of *ϕ*, the TDL cycles through a set of calibration gases, and the infrared gas analyzer (IRGA) reference and leaf chamber. The TDL remains on each sample for a period of *c*. 30 s, thus allowing a single measurement every *c*. 120–360 s, precluding continuous monitoring of the leaf chamber. Recently, Sakoda *et al*. (2021) and Liu *et al*. (2022) estimated mesophyll conductance in C_3_ plants through induction using the steady‐state TDL method and were only able to measure *c*. 15 data points over a 30 min activation curve.

Here, we developed an experimental design that measures *ϕ* every *c*. 10 s over a 30 min induction. Based on our previous metabolic modeling (Wang *et al*., 2021) we hypothesized that leakiness will be higher during activation of C_4_ photosynthesis than during steady‐state conditions. The hypothesis is tested directly here from near‐continuous Δ^13^C discrimination measurements through induction of photosynthesis in maize and sorghum.

## Materials and Methods

### Plant material and growth conditions

Sorghum (*Sorghum bicolor* L. Moench, Tx430) and maize (*Zea mays* L., B73) plants were grown in a controlled‐environment glasshouse at the University of Illinois at Urbana‐Champaign. Temperature in the glasshouse was 28°C : 24°C, day : night. Plants were grown in 20 l pots filled with peat‐and‐perlite growing medium (BM6; Berger, Saint‐Modeste, QC, Canada). Measurements were taken on plants at 40 d after planting. Plants were kept in darkness for ≥ 30 min before measurement. The youngest fully expanded leaf on the main stem, as indicated by a fully emerged ligule, was selected for enclosure into the controlled‐environment measurement chamber.

### Gas‐exchange measurements

For sorghum, the leaf was placed in the opaque conifer chamber (LI‐6400‐22; Li‐Cor Environmental, Lincoln, NE, USA) with an integrated RGB light source (LI‐6400‐18; Li‐Cor Environmental) attached to a LI‐6400XT gas‐exchange system (Li‐Cor Environmental). The chamber was fitted with a leaf thermocouple (Omega Engineering Inc., Norwalk, CT, USA) (Fig. S1a). To minimize leakage from the chamber, an opaque flexible polymer sealant (Qubitac Sealant; Qubit Systems Inc., Kingston, ON, Canada) was applied around the chamber lips after enclosing the leaf (Fig. S1a). For maize, the leaf was placed in the large leaf and needle chamber (LI‐6800‐13; Li‐Cor Environmental) incorporating the large light source (LI‐6800‐03; Li‐Cor Environmental) (Fig. S1b). The flows to the reference and sample IRGAs were monitored to ensure that both analyzers received sufficient flow. Because maize has a large midvein, the sample chamber pressure was set to 0.1 kPa to ensure that any leaks were out of, not into, the sample chamber. The average (±SE) leakage from the chamber from all the maize measurements was 6.4 ± 1.9 μmol s^−1^, which accounted for 2.1 ± 0.68% of the flow. For both species, the leaf was placed in the chamber in darkness with a leaf temperature of 27°C, CO_2_ reference of 800 μmol mol^−1^, an [O_2_] of 21% and a flow rate of 300 μmol s^−1^. We controlled reference [CO_2_] to avoid artifacts caused by system adjustment. Reference CO_2_ of 800 μmol mol^−1^ was used to ensure that the sample [CO_2_] during the measurement is not lower than the ambient CO_2_. Leaf area was calculated as the product of the internal length of the chamber and the average of the width of the leaf at both ends of the chamber.

### Isotopic gas‐exchange measurement

The gas‐exchange system was coupled to a TDL (model TGA 200A; Campbell Scientific Inc., Logan, UT, USA) to measure [^12^CO_2_], [^13^CO_2_] and δ^13^C (Bowling *et al*., 2003; Pengelly *et al*., 2010; Ubierna *et al*., 2013; Jaikumar *et al*., 2021). For sorghum, the reference line for the LI‐6400XT was split on the back of the sensor head so that a portion of the reference gas was diverted to the TDL. The exhaust gas from the leaf chamber was taken from the match port on the chamber, fitted with a three‐way valve to allow the gas to go to either the TDL or the match valve on the LI‐6400XT (Fig. S1a). For maize, the TDL was connected to the LI‐6800 reference air stream using the reference port on the back of sensor head while the port on the front of the head supplied air from the leaf chamber (Jaikumar *et al*., 2021; Fig. S1b). CO_2_‐free air (N_2_/O_2_) with a known [O_2_] was created by mixing two gas streams using precision mass flow controllers (Omega Engineering Inc.). A portion of this N_2_/O_2_ air traveled to the gas‐exchange system while the remainder was used as CO_2_‐free air in calibration to correct for drift in the TDL over the course of the measurements. The TDL was calibrated using the concentration series method by diluting a 10% CO_2_ gas cylinder into the N_2_/O_2_ stream to produce three different [CO_2_] of the same isotopic composition (Pengelly *et al*., 2010; Tazoe *et al*., 2011; Ubierna *et al*., 2013; Jaikumar *et al*., 2021). The measurement sequence cycled through eight gas streams in the following sequence: CO_2_‐free air, followed by three different CO_2_ concentrations of the same isotopic signature, air from a calibration tank with a known [^12^CO_2_], [^13^CO_2_], and δ^13^C composition (NOAA Global Monitoring Laboratory, Boulder, CO, USA), the IRGA reference and leaf chamber air streams, and the IRGA reference again. Each step had a duration of 20 s, except for the leaf chamber air, which had a duration of 600 s with a total cycle time of 740 s. Measurements were collected at a 10 Hz interval and averaged over 10 s as a single data point. The first 10 s of each gas stream was excluded to produce a single data point, except for the sample line which produced 59 data points each cycle. Instrument performance, including Allan deviations and instrument precision, are presented in Notes S1.

When the TDL switched to measuring the gas from the leaf chamber, the irradiance incident on the leaf was changed from 0 to 1800 μmol quanta m^−2^ s^−1^. The gas‐exchange system was set to auto‐log at 10 s intervals over the course of 30 min. Dark respiration rate was recorded before illumination.

### Calculations of photosynthetic discrimination (Δ^13^C) and leakiness (*ϕ*)

Instantaneous online determination of observed photosynthetic discrimination (Δ^13^C_obs_; Table 1) was calculated according to Evans & von Caemmerer (2013):

(Eqn 1) $$\Delta^{13}C_{\mathrm{obs}}=\frac{1000\xi \left(\delta^{13}C_{\text{samp}}-\delta^{13}C_{\mathrm{ref}}\right)}{1000+\delta^{13}C_{\text{samp}}-\xi \left(\delta^{13}C_{\text{samp}}-\delta^{13}C_{\mathrm{ref}}\right)}$$

where δ^13^C_samp_ and δ^13^C_ref_ are the carbon isotope compositions of the leaf chamber and reference air, respectively, and *ξ* is:

(Eqn 2) $$\xi =\frac{C_{\mathrm{ref}}}{C_{\mathrm{ref}}-C_{\text{samp}}}$$

*C*_ref_ and *C*_samp_ are the [CO_2_] of dry air entering and exiting the leaf chamber, respectively, as measured by the TDL. For each measurement sequence, we averaged the [CO_2_] and δ^13^C of the reference air measured before and after the measurement of leaf chamber air.

**Table 1:** List of symbols used in the text for calculating leakiness in maize and sorghum.

Variable	Definition	Units	Equations/value/reference
*a*	Fractionation across the stomata	‰	4.4 (Craig, 1953; *a*_*s*_ in Ubierna *et al*., 2013, 2018)
*a*_b_	Fractionations across the boundary layer	‰	2.9
$\bar{a}$	Weighted fractionation across the boundary layer and stomata in series	‰	Eqn 18 (Ubierna *et al*., 2013, 2018)
*Α*	Rate of photosynthesis	μmol m^−2^ s^−1^	Measured
*b*_3_	^13^C fractionation during carboxylation by Rubisco, including respiration and photorespiration fractionations	‰	Eqn 13 (Farquhar, 1983)
$b_{3}^{\prime }$	^13^C fractionation during carboxylation by Rubisco	‰	30
*b*_4_	Net fractionation by CO_2_ dissolution, hydration and phosphoenolpyruvate carboxylase (PEPC) including respiratory fractionation	‰	Eqn 14 (Farquhar, 1983)
$b_{4}^{\prime }$	Net fractionation by CO_2_ dissolution, hydration and PEPC activity dependent upon temperature	‰	Eqn 15
*C*_a_	Ambient CO_2_ partial pressure	Pa	Measured in μmol mol^−1^ air
*C*_bs_	CO_2_ partial pressure in the bundle‐sheath cells	Pa	Eqn 7
*C*_i_	CO_2_ partial pressure at the intercellular airspace	Pa	Measured in μmol mol^−1^ air
*C*_s_	CO_2_ partial pressure at the leaf surface	Pa	Measured in μmol mol^−1^ air
*C*_ref_	CO_2_ concentration of the dry air exiting the leaf chamber	μmol mol^−1^	Measured
*C*_samp_	CO_2_ concentration of the dry air exiting the leaf chamber	μmol mol^−1^	Measured
*e*	^13^C fractionation during decarboxylation	‰	0 (Evans & von Caemmerer, 2013; Ubierna *et al*., 2013)
*e*′	^13^C fractionation during decarboxylation including the effect of a respiratory substrate isotopically distinct from recent photosynthate	‰	Eqn 16
*E*	Rate of transpiration	mol m^−2^ s^−1^	Measured
*f*	^13^C fractionation during photorespiration	‰	1.6‰ (Ubierna *et al*., 2013)
$g_{\mathrm{ac}}^{t}$	Total conductance to CO_2_ diffusion including boundary layer and stomatal conductance	mol m^−2^ s^−1^	Measured
*g*_bs_	Bundle‐sheath conductance to CO_2_	mol m^−2^ s^−1^	0.00113 (Brown & Byrd, 1993)
*J*_t_	Total electron transport rate	μmol m^−2^ s^−1^	Eqn 3 (von Caemmerer, 2000)
*O*_m_	O_2_ partial pressure in the mesophyll cells	Pa	21.2 Pa atmospheric pressure
*O*_s_	O_2_ partial pressure in the bundle‐sheath cells	Pa	Eqn 11 (von Caemmerer, 2000)
*R*_d_	Leaf mitochondrial respiration in the light assumed to equal the rate of respiration in the dark	μmol m^−2^ s^−1^	Measured
*R*_m_	Rate of mesophyll cell respiration in the light	μmol m^−2^ s^−1^	*R*_m_ = 0.5*R*_d_
*s*	Fractionation during leakage from the bundle‐sheath cells	‰	1.8 (Henderson *et al*., 1992)
*t*	Ternary effect	‰	Eqn 17
*V*_c_	Rubisco carboxylation rate	μmol m^−2^ s^−1^	Eqn 9 (von Caemmerer, 2000)
*V*_o_	Rubisco oxygenation rate	μmol m^−2^ s^−1^	Eqn 10 (von Caemmerer, 2000)
*V*_p_	PEP carboxylation rate	μmol m^−2^ s^−1^	Eqn 8 (von Caemmerer, 2000)
*x*	Fraction of *J*_t_ allocated to the C_4_ cycle		0.4 (von Caemmerer, 2000)
*α*	Fraction of PSII activity in the bundle sheath		0 (von Caemmerer, 2000)
δ^13^C_gatm_	Isotopic signature of growth CO_2_	‰	−8
δ^13^C_ref_	Isotopic signature of the CO_2_ entering the leaf chamber	‰	Measured
δ^13^C_samp_	Isotopic signature of the CO_2_ exiting the leaf chamber	‰	Measured
*ξ*	Ratio of the ^12^CO_2_ mole fraction in the dry air coming into the gas‐exchange cuvette over the difference in ^12^CO_2_ mole fractions of air in and out of the cuvette	unitless	Eqn 2
Δ^13^C_obs_	Observed ^13^C photosynthetic discrimination	‰	Eqn 1
*ϕ*_is_	Leakiness estimated assuming infinite mesophyll conductance	Unitless	Eqn 12

The electron transport flux (*J*_t_) was calculated as (von Caemmerer, 2000; Ubierna *et al*., 2013):

(Eqn 3) $$J_{t}=\frac{-\mathrm{II}+\sqrt{{\mathrm{II}}^{2}-4\cdot \mathrm{III}\cdot I}}{2\cdot \mathrm{III}}$$

where

(Eqn 4) $$I=\left(1+\frac{R_{d}}{A}\right)\left(R_{m}-g_{\mathrm{bs}}C_{m}-\frac{7g_{\mathrm{bs}}\gamma^{*}O_{m}}{3}\right)+\left(R_{d}+A\right)\left(1-\frac{7\alpha \gamma^{*}}{3\times 0.047}\right)$$

(Eqn 5) $$\mathrm{II}=\frac{1-x}{3}\left[\frac{g_{\mathrm{bs}}}{A}\left(C_{m}-\frac{R_{m}}{g_{\mathrm{bs}}}-\gamma^{*}O_{m}\right)-1-\frac{\alpha \gamma^{*}}{0.047}\right]-\frac{x}{2}\left(1+\frac{R_{d}}{A}\right)$$

(Eqn 6) $$\mathrm{III}=\frac{x-x^{2}}{6A}$$

*R*_d_ is leaf mitochondrial respiration in the light, assumed to be equal to dark respiration, *R*_m_ (*R*_m_ = 0.5*R*_d_) is the rate of mesophyll cell respiration in the light, and *A* is the rate of net CO_2_ assimilation. *C*_m_ is CO_2_ concentration in the mesophyll cells, which was assumed to equal measured *C*_i_, *γ* ^*^ is half of the reciprocal of Rubisco specificity (0.000193; von Caemmerer *et al*., 1994), *O*_m_ is the O_2_ mol fraction in the mesophyll cells (210 000 μmol mol^−1^), and *x* is the portion of ATP used by the C_4_ cycle, assumed to equal 0.4 (von Caemmerer, 2000). The fraction of PSII activity in the bundle sheath (*α*) was assumed to be 0 for maize and sorghum (von Caemmerer, 2000). The bundle‐sheath conductance to CO_2_ (*g*_bs_) was set as 0.00113 mol m^−2^ s^−1^ (Brown & Byrd, 1993).

We calculated the CO_2_ partial pressure in the bundle‐sheath cells (*C*_s_), PEP carboxylation rate (*V*_p_), Rubisco carboxylation rate (*V*_c_), oxygenation rate (*V*_o_) and the O_2_ partial pressure in the bundle‐sheath cells (*O*_s_) using the following expressions (von Caemmerer, 2000):

(Eqn 7) $$C_{\mathrm{bs}}=\frac{\gamma^{*}O_{s}\;\left[\frac{7}{3}\left(A+R_{d}\right)+\frac{\left(1-x\right)J_{t}}{3}\right]}{\frac{\left(1-x\right)J_{t}}{3}-\left(A+R_{d}\right)}$$

(Eqn 8) $$V_{p}=\frac{xJ_{t}}{2}$$

(Eqn 9) $$V_{c}=\frac{A+R_{d}}{1-\frac{\gamma^{*}O_{s}}{C_{\mathrm{bs}}}}$$

(Eqn 10) $$V_{o}=\frac{V_{c}-A-R_{d}}{0.5}$$

(Eqn 11) $$O_{s}=\frac{\mathrm{\alpha A}}{0.047g_{\mathrm{bs}}}+O_{m}$$

We estimated leakiness, assuming infinite mesophyll conductance, using the model proposed by Ubierna *et al*. (2013):

(Eqn 12) $$\phi_{\text{is}}=\frac{C_{\mathrm{bs}}-C_{i}}{C_{i}}\frac{\left(1-t\right)\Delta^{13}C_{\mathrm{obs}}C_{a}-a^{\prime }\left(C_{a}-C_{i}\right)-\left(1+t\right)C_{i}b_{4}}{\left(1+t\right)\left[b_{3}C_{\mathrm{bs}}-s\left(C_{\mathrm{bs}}-C_{i}\right)\right]+a^{\prime }\left(C_{a}-C_{i}\right)-\left(1-t\right)\Delta^{13}C_{\mathrm{obs}}C_{a}}$$

where *C*_a_, and *C*_i_ are the ambient and intercellular CO_2_ partial pressures, respectively, and *t* is the ternary effect (Farquhar & Cernusak, 2012). The fractionation during leakage from the bundle‐sheath cells (*s*) is 1.8‰, and *b*_3_ and *b*_4_ were defined as (Farquhar, 1983):

(Eqn 13) $$b_{3}=b_{3}^{\prime }-\frac{e^{\prime }R_{d}}{V_{c}}-\frac{fV_{o}}{V_{c}}$$

(Eqn 14) $$b_{4}=b_{4}^{\prime }-\frac{e^{\prime }R_{m}}{V_{p}}$$

where *f* is fractionation during photorespiration, assumed to be 11.6‰ (Lanigan *et al*., 2008). $b_{3}^{\prime }$ (30‰) is Rubisco fractionation, and $b_{4}^{\prime }$, the net fractionation by CO_2_ dissolution, hydration and PEPC activity at 27°C, was calculated according to Mook *et al*. (1974), which is used by Henderson *et al*. (1992) and von Caemmerer *et al*. (2014):

(Eqn 15) $$b_{4}^{\prime }=\frac{-9.483\times 1000}{273+\left(T{}^{\circ}C\right)}+23.89+2.2$$

We estimated *e*′, which is the ^13^CO_2_ fractionation during decarboxylation and takes into account respiration that is isotopically distinct from recent photosynthate, as previously discussed (Wingate *et al*., 2007; Ubierna *et al*., 2018):

(Eqn 16) $$e^{\prime }=e+\delta^{13}C_{\mathrm{ref}}-\delta^{13}C_{\text{gatm}}$$

where *e* is the respiratory fractionation during decarboxylation, 0‰, δ^13^C_gatm_ is the isotopic signature of the CO_2_ in the air where the plants were grown, assumed to be −8‰, and δ^13^C_ref_ is the isotopic signature of the measurement CO_2_ and was between −10‰ and −6.5‰.

The ternary effect (*t*) (Farquhar & Cernusak, 2012) takes into account the effect of transpiration on the rate of CO_2_ assimilation through the stomata and is calculated as:

(Eqn 17) $$t=\frac{\left(1+a^{\prime }\right)E}{2g_{\mathrm{ac}}^{t}}$$

where *E* is the rate of transpiration, $g_{\mathrm{ac}}^{t}$ is the total conductance to CO_2_ diffusion from the atmosphere to the intercellular airspace including boundary layer and stomatal conductance (von Caemmerer & Farquhar, 1981), and a' denotes the combined fractionation factor through the leaf boundary layer and the stomata:

(Eqn 18) $$a'=\frac{a_{b}\left(C_{a}-C_{s}\right)+a\left(C_{s}-C_{i}\right)}{C_{a}-C_{i}}$$

where *C*_s_ is the leaf surface CO_2_ partial pressure, *a*_b_ (2.9‰) is the fractionation occurring through diffusion in the boundary layer, and *a* (4.4‰) is the fractionation as a result of diffusion in air (Craig, 1953).

### The error associated with Δ^13^C_obs_ measurements

The error associated with Δ^13^C_obs_ was calculated according to Ubierna *et al*. (2018):

(Eqn 19) $$\text{Error}=\sqrt{2}\;\mathrm{\xi X}$$

where *X* is instrument precision (Notes S1). The error (%) was calculated as $\text{error}\left(\%\right)=\left(\text{error}/\Delta^{13}C_{\mathrm{obs}}\right)\times 100.$

Instrument precisions during the measurements were 0.24‰ and 0.14‰ for sorghum and maize, respectively. We excluded all data points where the error in Δ^13^C_obs_ was > 50%. This occurred in the first 144 s for sorghum and the first 110 s for maize.

### Data processing

A fully automatic data processing and leakiness calculation tool was developed in Matlab. The tool used the pretreated (LI‐6400XT and LI‐6800) data files and the raw TDL data to calculate the leakiness through the photosynthetic induction, with the equations described earlier. The TDL data were averaged every 10 s to match the gas‐exchange data and to reduce noise (Fig. S2). See the Data availability statement for access to this tool.

### Correction of the system delay

System delays were caused by both the large volume of the leaf chambers and the gas path from leaf chamber to the TDL (see Methods S1 for further information). The time delay from leaf chamber to the TDL was estimated by pulsing the leaf chamber with high CO_2_ and monitoring the time it took to observe the CO_2_ spike in the TDL. A 5‐cm‐wide paper strip was clipped into the chamber sealed with opaque flexible polymer sealant (Qubitac Sealant) to mimic the effect of the leaf on flow and mixing. The [CO_2_] was recorded every 2 s until the chamber outlet [CO_2_] was stable at 400 μmol mol^−1^ (Fig. S3). Three different flow rates were measured, 300, 500 and 700 μmol s^−1^. For each flow rate, the measurements were repeated three times. Results were used to estimate the chamber volume (*V*_chamber_) and time constant (*τ*), as defined later (https://www.licor.com/env/support/LI‐6400/topics/custom‐chamber.html).

Assuming the gas is well mixed in the chamber, for an open, flow‐through system, the [CO_2_] in the chamber *C*(*t*) at time *t* is:

(Eqn 20) $$C\left(t\right)=C_{\text{in}}-\left(C_{\text{in}}-C_{0}\right)e^{\frac{-\mathrm{tf}V_{m}}{V_{\text{chamber}}}}$$

where *C*_0_ is the initial chamber [CO_2_], *C*_in_ is the incoming [CO_2_], *V*_m_ is the molar volume of air, which was assumed to approximate an ideal gas at standard atmospheric pressure and 27°C, and is set as 24.6 l mol^−1^, *f* is the air flow rate (s) and *V*_chamber_ is the chamber volume (l). Then, an ordinary differential equation model was used to estimate the system delay during photosynthetic induction measurement:

(Eqn 21) $$\frac{dC}{dt}=\frac{S_{\text{leaf}}\left(A_{\text{leaf}}\left(C\right)-A_{\text{leaf}}^{\prime }\right)}{V_{\text{chamber}}}\;V_{m}$$

where *S*_leaf_ is the leaf area, *A*_leaf_ (*C*) is the leaf carbon assimilation rate estimated by the gas‐exchange system at a given [CO_2ref_], and $A_{\text{leaf}}^{\prime }$ is the actual carbon assimilation rate. Here we set it as:

(Eqn 22) $$A_{\text{leaf}}^{\prime }=A_{f}\left(1-e^{-\frac{t}{\tau_{A}}}\right)$$

where *A*_f_ is the steady‐state photosynthesis rate at high light, *τ*_A_ is the time constant of the induction of photosynthesis, and *A*_f_ and *τ*_A_ were set as 40 μmol m^−2^ s^−1^ and 300 s, respectively, according to gas‐exchange measurements.

### Rubisco activation estimation

If the photosynthetic rate is limited by Rubisco, the maximum Rubisco activity is:

(Eqn 23) $$V_{\text{cmax}}=A+R_{d}$$

Thus, the rate constant of Rubisco activation is equal to the rate constant of induction of CO_2_ assimilation. A semilogarithmic plot of the difference between *A* and steady‐state CO_2_ assimilation at 1800 μmol m^−2^ s^−1^ (*A*_f_) as a function of time during photosynthetic induction was plotted (Fig. S4). The linear portion of the semilogarithmic plot reflects an exponential phase in the time course that is proposed to be limited primarily by Rubisco (Woodrow & Mott, 1993; Wang *et al*., 2021). The slope of this linear portion is equal to the negative reciprocal of the time constant for CO_2_ assimilation and Rubisco activation (*τ*_A_ *= τ*_Rubisco_). As Rubisco limits the later phase of the induction of C_4_ crops, we used the measured photosynthetic rate between 300 and 900 s for this estimation.

### Statistical analyses

Normal distribution and homogeneity of variances were tested by the Shapiro–Wilk and Levene tests, respectively. Student's *t*‐test was used to determine if the means of two datasets were significantly different from each other (*P* < 0.05). All statistical analyses used Python (v.3.7), Shapiro–Wilk test, Levene test and Student's *t*‐test were performed using the SciPy library. The piecewise function was fitted by linear and exponential goodness‐to‐fit regression (OriginPro v.2020; OriginLab, Northampton, MA, USA).

## Results

### Leakiness during photosynthetic induction in sorghum

**Fig. 1:**
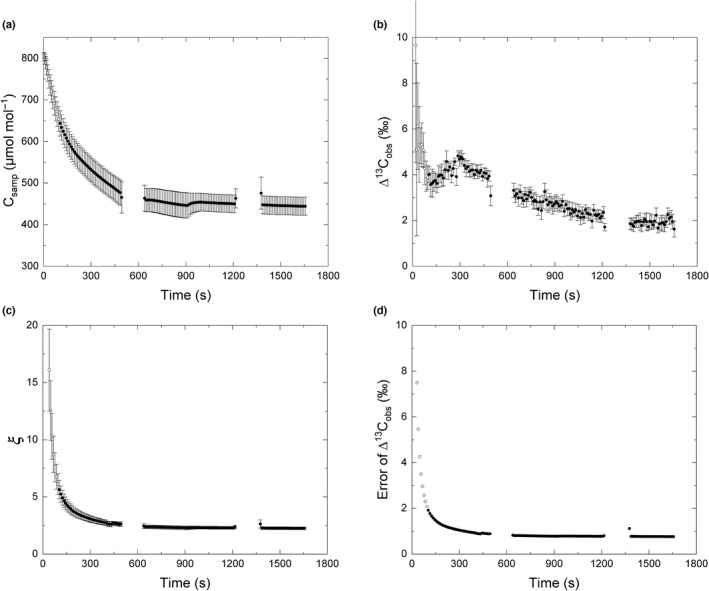
Measured carbon isotope discrimination during photosynthetic induction of sorghum (Tx430) using a tunable diode laser absorption spectroscope (TDL) coupled to a gas‐exchange system (LI‐6400XT). (a) Sample [CO_2_]; (b) the observed leaf photosynthetic discrimination (Δ^13^C_obs_); (c) *ξ*, an estimate of the uncertainty in Δ^13^C_obs_ and *ϕ* calculations; (d) error of Δ^13^C_obs_ going from dark to high light (1800 μmol m^−2^ s^−1^). Time 0 s refers to when the light was switched on. Open dots represent the data points where the error of Δ^13^C_obs_ was > 50%. The TDL was calibrated after every 600 s of measurement. The gas from leaf chamber was not measured during the calibration and the measurement of reference gas (140 s), which occurred from *c*. 490 to 640 s and *c*. 1215 to 1365 s. Leaf gas‐exchange and carbon discrimination of the youngest fully expanded leaf was measured on 40‐d‐old sorghum (Tx430) plant. The leaf was dark‐adapted for 30 min before the measurement. Each data point is the mean (±SE) of eight plants (*n* = 8).

During the photosynthetic induction, sample [CO_2_] declined rapidly from 820 μmol mol^−1^ to a steady state of *c*. 450 μmol mol^−1^ at *c*. 600 s (Fig. 1a). Photosynthetic discrimination (Δ^13^C_obs_) was used to estimate leakiness (*ϕ*), declining from an initial 10‰ to *c*. 3.5‰ at 120 s, then rising to 5‰ at 300 s and finally declining to a steady state of *c*. 2.0‰ at *c*. 1500 s (Fig. 1b). As expected, *ξ*, a measure of the uncertainty in Δ^13^C_obs_ was high (15) when rates of photosynthesis were low and decreased as *A* increased through induction, to a steady state of 2.5 at *c*. 600 s (Fig. 1c). The error of Δ^13^C_obs_ was higher than 2‰ (50% of Δ^13^C_obs_) in the first 100 s of the measurement and quickly declined to around 1.2‰ (30% of Δ^13^C_obs_) by 200 s (Fig. 1d; Table N1 in Notes S1).

**Fig. 2:**
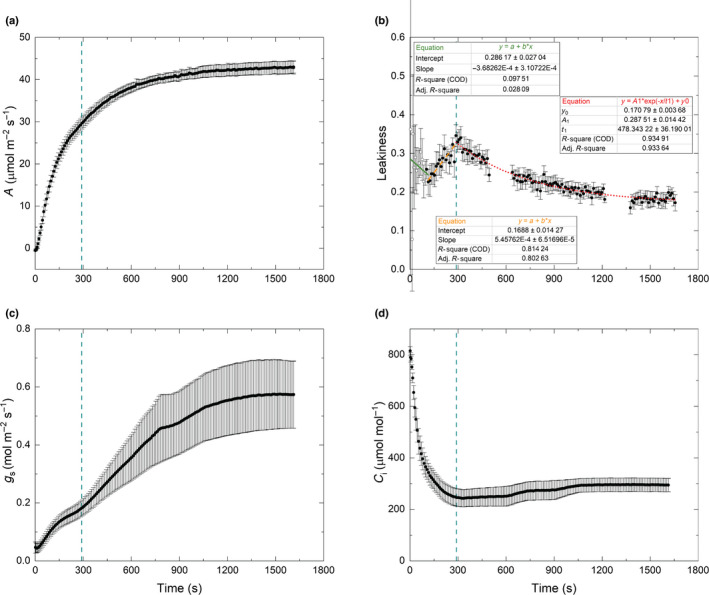
CO_2_ assimilation and bundle‐sheath leakiness during photosynthetic induction of sorghum measured with an LI‐6400XT coupled to a tunable diode laser absorption spectroscope (TDL). (a) CO_2_ assimilation rate (*A*). (b) Bundle‐sheath leakiness (*ϕ*, Eqn 12). Open dots represent the data points derived from values of observed discrimination that had large uncertainty (the error in the calculated Δ^13^C_obs_ was > 50% of its calculated value). (c) Stomatal conductance to water vapor (*g*_s_). (d) Intercellular CO_2_ concentration (*C*_i_). The dotted vertical lines mark the time of highest leakiness, which was 286 s. *t*_1_ is the time constant (*τ*) of the exponential curve for leakiness. Time 0 s is when the light was switched on to 1800 μmol m^−2^ s^−1^. Each data point is the mean (±SE) of eight plants (*n* = 8).

In the first 120 s in high light (1800 μmol quanta m^−2^ s^−1^), leakiness (*ϕ*) in sorghum declined from 0.32 to *c*. 0.23; however, *ϕ* then increased to *c*. 0.35 at 300 s, before gradually decreasing and reaching a steady state of *c*. 0.18 (Fig. 2b) at *c*. 1500 s. The leakiness curve was fitted with a piecewise function. No obvious trend was found in the first segment (*R*‐squared (*R* ^2^) = 0.098; Fig. 2b), which is also the segment with the greatest error of Δ^13^C_obs_. The second segment of the piecewise function showed linear growth (*R* ^2^ = 0.81); during this time the error rapidly declined to < 50% of the associated measurement. The third segment was exponential decline (*R* ^2^ = 0.93; Fig. 2b). The transition time point of the leakiness curve of sorghum occurred at *c*. 290 s. Excluding the initial 100 s of measurement, given its high error of Δ^13^C_obs_, the average (±SE) *ϕ* was 0.237 ± 0.012 over the 1500 s period of induction, which was 32% higher than the steady‐state *ϕ* in high light (0.180 ± 0.015, *P* = 0.005; Fig. 4a (see later); Table S1), indicating a substantial loss of efficiency during induction, compared with the steady state. The average *ϕ* value over the first 600 s period of induction was 0.289 ± 0.022, which was 61% higher than the steady‐state *ϕ* (*P* < 0.001; Fig. 4a (see later); Table S1). The reference [CO_2_] was set as 800 μmol mol^−1^ to minimize the limitations induced by stomatal and mesophyll conductance of CO_2_ to PEPC. In sorghum, intercellular [CO_2_] (*C*_i_) was always > 200 μmol mol^−1^, and so assumed not to be limiting to PEP carboxylation (Fig. 2d). Stomatal conductance to water vapor increased from 0.04 to *c*. 0.57 mol m^−2^ s^−1^ through the induction (Fig. 2c). The time constant of photosynthetic induction (τ_A_) between 300 and 900 s was 332 s, which was assumed to reflect the kinetics of Rubisco activation (Eqn 21; Fig. S4).

### Leakiness during photosynthetic induction in maize

**Fig. 3:**
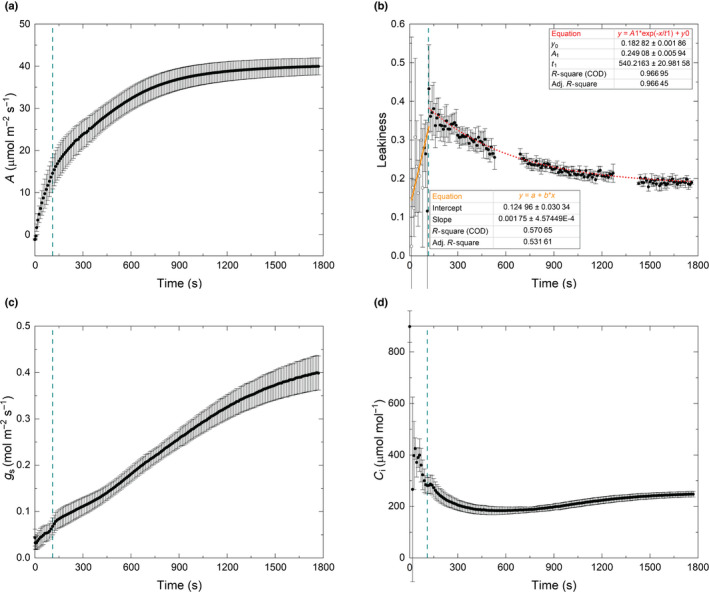
CO_2_ assimilation and bundle‐sheath leakiness during photosynthetic induction of maize B73 measured with an LI‐6800 coupled to a tunable diode laser absorption spectroscope (TDL). (a) CO_2_ assimilation rate (*A*). (b) Bundle‐sheath leakiness (*ϕ*, Eqn 12). Open dots represent data points derived from values of observed discrimination that had large uncertainty (the error in the calculated Δ^13^C_obs_ was > 50% of its calculated value). (c) Stomatal conductance to water vapor (*g*_s_); and (d) intercellular CO_2_ concentration (*C*_i_). The dotted vertical lines mark the time of highest leakiness, which was 110 s. *t*_1_ is the time constant (*τ*) of the exponential curve for leakiness. Time 0 is when the light was switched on to 1800 μmol m^−2^ s^−1^. The TDL was calibrated after every 600 s of measurement. Each point is the mean (±SE) of six plants.

**Fig. 4:**
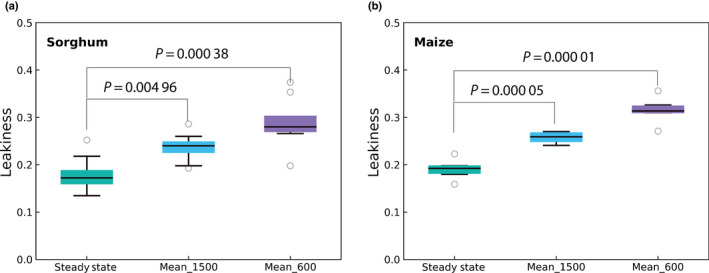
The comparison between steady‐state and transient leakiness in sorghum (a) and maize (b). Steady state, the average leakiness after 1500 s; Mean_1500, the average leakiness over the 1500 s period of induction; Mean_600, the average leakiness of the first 600 s in the induction. The data points with the error of Δ^13^C_obs_ > 50% were excluded. Data of each replicate were listed in Tables S1, S2. *P*‐values were calculated using Student's *t*‐test. Black circles represent the outliers; black lines in boxes show the medians. Upper and lower whiskers represent the maximum and minimum values, respectively.

During the dark to high‐light transition, leakiness increased faster in maize than in sorghum (Figs 3b, S8; see later). Similar to the measurement of sorghum, the error associated with Δ^13^C_obs_ estimation was > 50% for the first 90 s (Fig. S5d open circles) and photosynthetic discrimination rose rapidly to *c*. 120 s before a slow decrease to the steady state (Fig. S5b). As with sorghum, *ξ* was high when rates of photosynthesis were low and decreased with increasing rates of assimilation to a steady state at *c*. 600 s (Fig. S5c). *ϕ* during the photosynthetic induction in maize was fitted with the piecewise function. The first segment of the piecewise function was linear growth (Fig. 3b). The first segment of the piecewise function was linear increase, and *R* ^2^ of the linear regression was 0.57; however, the error of Δ^13^C_obs_ in this segment is large (Fig. S5d). After 110 s, *ϕ* during the induction can be fitted with an exponential decline function (Fig. 3b). The time constant of the exponential decline in *ϕ* was 540 s. The highest *ϕ* was *c*. 0.4 at 110 s. Excluding the initial 90 s of measurement, given its high error of Δ^13^C_obs_, over the 1500 s period of induction, the average *ϕ* was 0.258 ± 0.006, which was 35% higher than the steady‐state *ϕ* at high light (0.191 ± 0.010). Average *ϕ* over the first 600 s period of induction was 0.315 ± 0.014, which was 65% higher than the steady state *ϕ* (*P* < 0.001; Fig. 4b; Table S2). As in the case of sorghum, intercellular [CO_2_] (*C*_i_) of maize was always > 200 μmol mol^−1^ (Fig. 3d). Stomatal conductance to water vapor increased from 0.02 to about 0.4 mol m^−2^ s^−1^ through induction (Fig. 3c).

**Fig. 5:**
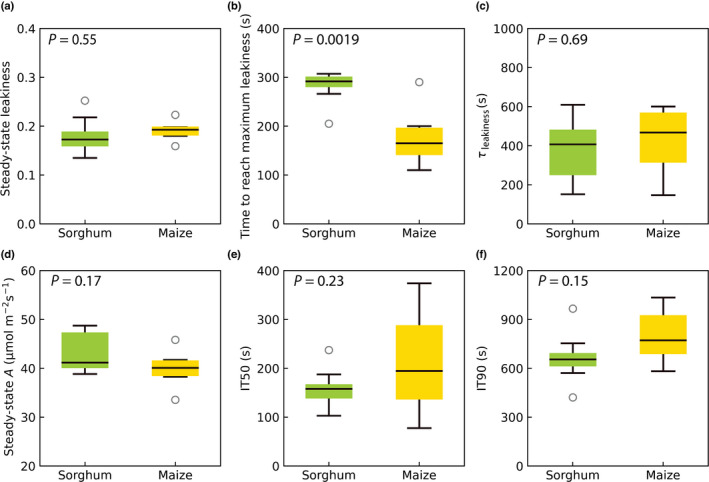
Mean and variation of steady‐state leakiness, the time to reach the maximum leakiness and the time constant of the exponential decay (*τ*_leakiness_), steady‐state CO_2_ assimilation rate (*A*), and IT50 and IT90 during the induction in sorghum and maize. (a) Average leakiness after 1500 s; (b) the time at the end of the linear growth segment of leakiness; (c) τ_leakiness_, the time constant of exponential decline segment; (d) average *A* after 1500 s; (e) IT50, the time at which *A* reached 50% of the steady state; (f) IT90, the time at which *A* reached 90% of the steady state *A. P*‐values were calculated using Student's *t*‐test. Black circles represent the outliers; black lines in boxes show the medians; upper and lower whiskers represent the maximum and minimum values, respectively.

After 1800 s (30 min) in photosynthetic photon flux density of 1800 μmol m^−2^ s^−1^, maize and sorghum had similar rates of steady‐state CO_2_ assimilation and leakiness (Fig. 5a,d), and there was no significant difference between the species in the time taken for *A* to reach 50% and 90% of the steady‐state value, IT50 and IT90, respectively (Fig. 5e,f). However, the rise in leakiness in sorghum was significantly more prolonged than in maize, as indicated by the time taken to reach the peak of leakiness during induction (Fig. 5b). The speed of exponential decay of *ϕ* was similar, and there was no significant difference between the two species in *ϕ* (Fig. 5c), which is the time constant of exponential decline segment of the leakiness function (Figs 2b, 3b; curve‐fitting parameter *t*_1_).

## Discussion

### Coordination between the C_3_ and C_4_ cycles was disrupted during photosynthetic induction

Coordination between the C_3_ and C_4_ cycles is essential to the high efficiency of C_4_ photosynthesis. We estimated CO_2_ leakiness with stable carbon isotopes by coupling a TDL to a gas‐exchange system. Leakiness (*ϕ*) is the proportion of CO_2_ released by decarboxylation of dicarboxylates in the bundle sheath that leaks back to the mesophyll. Any variation in *ϕ* reflects the degree of coordination between the two cycles.

A complete metabolic model of NADP‐ME photosynthesis, incorporating activation of enzymes, stomatal induction and dynamic changes in metabolic pools predicted poor coordination and transient increase in *ϕ* during photosynthetic induction, as a result of a more rapid activation of PPDK in the mesophyll by the PPDK regulatory protein, than activation of Rubisco in the bundle sheath by Rubisco activase (Wang *et al*., 2021). The transient increases in *ϕ* in both maize and sorghum observed here are fully consistent with this explanation.

Leaves in a crop canopy often face intense and rapid light changes (Zhu *et al*., 2004; Wang *et al*., 2020; Qiao *et al*., 2021). Over this 1500 s period of induction, the average *ϕ* was > 30% higher than the steady‐state *ϕ* at high light for sorghum and maize. Leakiness over the first 600 s was 61% higher than the steady‐state *ϕ* for sorghum and 65% for maize. The lack of coordination between C_4_ and C_3_ cycles will substantially reduce the efficiency of C_4_ photosynthesis at both leaf and canopy levels. Although the present study uses an extreme case of fluctuation (i.e. an immediate transfer from darkness to full sunlight), leaves in the canopy will frequently experience transfer from 10% to full sunlight (Long *et al*., 2022). A recent application of a high‐throughput assay of Rubisco activation has shown that deactivation on transfer to shade is very rapid, occurring within a minute (Taylor *et al*., 2022). So why has natural selection not removed this inefficiency? In the wild, many C_4_ plants, including wild ancestors of maize and sorghum, are most abundant in hot semiarid and nutrient‐poor regions (De Wet, 1978; Yang *et al*., 2019). As a result, leaf canopies may be sparse, and cloud cover infrequent. In these conditions there will be fewer light fluctuations and little selective pressure to avoid these transient increases in *ϕ*. The dense modern crop canopies of maize and sorghum are recent in an evolutionary context, but here the losses as a result of these transient inefficiencies would be much greater.

### Differences of transient leakiness between sorghum and maize during induction

The induction rate of CO_2_ assimilation was similar between the two species, and a transient increase in leakiness was detected in both sorghum and maize (Figs 2a,b, 3a,b, S6). Leakiness reached a maximum significantly faster in maize than in sorghum (Fig. 5b), which most probably indicates faster activation of PPDK, possibly as a result of either more of its regulatory protein (PDRP) or a more efficient PDRP (Ashton *et al*., 1984; Burnell & Chastain, 2006; Wang *et al*., 2021). These results, consistent with the previous metabolic modeling of NADP‐ME C_4_ photosynthesis, through induction suggest activation of Rubisco as the key limitation through induction and the primary cause of lost efficiency. Rubisco activase (Rca) appears to be an exceptionally heat‐labile protein, implicated in loss of photosynthetic efficiency at high temperatures (Crafts‐Brandner & Salvucci, 2000). This implies that the loss of efficiency in these key crops would be amplified by rising global temperatures. This loss of efficiency might be overcome by breeding or engineering an increase in Rca content, and in particular more high‐temperature‐tolerant isoforms (Carmo‐Silva & Salvucci, 2013; Degen *et al*., 2021). Kim *et al*. (2021). These studies have shown that the redox‐regulated Rca‐α isoform is expressed in sorghum, sugarcane, maize and *Sateria* only at temperatures > 42°C and the time course of Rca‐α corresponds to recovery of Rubisco activation and the rate of photosynthesis from heat shock. However, overall variation in Rca in C_4_ crops has so far received little attention. Based on our estimation and previous studies, increasing the activity of Rca by either increasing Rca content or engineering a more efficient Rca would increase photosynthetic efficiency under constant and fluctuating light. Both now appear possible through bioengineering and possibly breeding (Long *et al*., 2022).

The high CO_2_ concentration supplied to the leaf chamber in our experiment (Figs 1a, S6a) minimized diffusional limitations (stomatal and mesophyll) to photosynthesis. During induction, the CO_2_ concentrations inside the leaf (*C*_i_) were > 200 and 180 μmol mol^−1^ for sorghum and maize, respectively (Figs 2d, 3d). Previous research (Wang *et al*., 2021) demonstrated that at ambient CO_2_ concentrations, slow stomatal opening during the middle phase of induction reduced both CO_2_ assimilation rate and leakiness in three C_4_ crops. The CO_2_ concentration used for measurements should have had a negligible effect on leakiness determined for the fast response of stomata in sorghum but could have impacted values for the slower response of stomata seen in maize. Mesophyll conductance (*g*_m_) could also be a limiting factor during induction. In this study, *g*_m_ was assumed to be infinite and constant. There are no experimental data on the variation of *g*_m_ during induction in C_4_ species. However, the main resistances to CO_2_ diffusion through, the cell wall and plasmalemma to the PEP carboxylase baring mesophyll cytoplasm, are probably unaffected by light, barring a Péclet effect with increasing outflow of water. This is a topic for subsequent investigation.

### Energy‐use efficiency of C_4_ crops under fluctuating light

The steady‐state *ϕ* values were *c*. 0.2 in maize and sorghum; thus 5.5 ATP are used to assimilate one CO_2_. However, over the 1500 s period of the induction, the average *ϕ* was 0.25. Moreover, the average *ϕ* of the first 600 s was *c*. 0.30 in both sorghum and maize. The higher transient *ϕ* will have increased the ATP consumption of assimilating a CO_2_ to 5.7 and 5.9, respectively. The energetic cost of CO_2_ assimilation is therefore higher in fluctuating light than under steady‐state conditions. However, when light is in excess, as in induction, this will have little effect.

Variation in carbon assimilation during fluctuating light was previously observed by Lee *et al*. (2022) across four NADP‐ME grass species and may well arise from variation in the degree to which the C_4_ and C_3_ cycles are coordinated, as was shown here for maize and sorghum, but was not determined in their study. Being able to estimate variation in *ϕ* between species and genotypes during fluctuating light will be necessary for developing strategies to improve C_4_ crop performance. Additionally, low‐growth‐light intensity increases steady‐state *ϕ* of shaded field‐grown *M*. × *giganteus* leaves, assuming the *C*_i_ in the bundle sheath is much higher than *C*_i_ in the *ϕ* estimation (Kromdijk *et al*., 2008). Although the underlying basis of the increased *ϕ* in shade‐adapted leaves may be different from the increased *ϕ* in fluctuating light, these leakages could be additive, which would further handicap the efficiency of leaves within C_4_ canopies. We demonstrated a new experimental design with the TDL to estimate *ϕ* at high resolution and under transient conditions. This technique provides opportunities to investigate further the underlying causes of increased *ϕ*, as well as facilitating strategies to improve C_4_ plant performance in fluctuating light.

### A new experimental design for the TDL with a gas‐exchange system

The coupling of a TDL with a gas‐exchange system has been used to measure leakiness in C_4_ plants under photosynthetic steady‐state conditions (Pengelly *et al*., 2010; Ubierna *et al*., 2011, 2013). Recent work has coupled gas‐exchange systems to a TDL to measure mesophyll conductance during induction curves (Sakoda *et al*., 2021; Liu *et al*., 2022); however, these studies were only able to estimate mesophyll conductance every 120 s over the activation curve. Our method, allowing the TDL to remain on the leaf chamber for 600 s, enabled us to have a nearly continuous high‐resolution (10 s) dataset over a 30 min high‐light induction. This allowed the measurement of *ϕ* under nonsteady‐state conditions. The stability and precision of the instrument are critical to the accuracy of the estimation of *ϕ* (Fig. N1 in Notes S1). The error of our laser could be controlled within a limited range during the experiment, and the averaging time of 10 s significantly reduced the system noise and improved the prediction accuracy, with sufficient time resolution for the purposes of the questions asked in this study (Notes S1, laser performance). In the first *c*. 100 s of the induction, the error associated with Δ^13^C_obs_ estimation was > 50%, which indicated that our measurements were masked by instrument error. Thus, we minimized our interpretation of the leakiness values in this time frame. The error associated with photosynthetic discrimination (Δ^13^C_obs_) was < 30% after 200 and 130 s for sorghum and maize, respectively, indicating that the error associated with the TDL was acceptable for the remainder of the induction. The error associated with the laser can change through time, environment and with retuning of the laser. These characters were verified for each laser and tested before each application. As CO_2_ concentration around Rubisco (*C*_bs_) should not be much higher than CO_2_ in mesophyll (*C*_m_) at the beginning of the induction, the complete calculation of leakiness (Eqn 12) was used instead of the simplified model that assumes the *C*_bs_ is much higher than *C*_m_ (Fig. S7). Additionally, we developed a program to calculate the leakiness automatically from raw carbon isotope and gas‐exchange data, which improved throughput of data analysis.

The accuracy of the measured gas‐exchange values was significantly improved by correcting for the time delay of the system (Figs S8, S9). The measuring noise of carbon isotope mole fractions was also constrained by averaging signals within every 10 s (Fig. N1 in Notes S1), and thus the noise in leakiness estimation was also reduced (Fig. S2), although the accuracy of the measurement was still limited by the precision of the gas‐exchange system and the TDL in the first *c*. 100 s after the illumination. We expect that our measurement experience and data‐processing program will help researchers to save time and develop new applications for this system.

## Author contributions

The project was conceived by YW, SSS and SPL. YW and SSS performed the experiments. YW developed the data‐processing tool and analyzed the data. SSS developed the method for continual monitoring of leakage through induction. CJB and SSS set up the TDL. YW, SSS and SPL wrote the manuscript with insights from DRO, RAB and CJB. YW and SSS contributed equally to this work.

## Supporting information


**Fig. S1** Pictures of the setup for the two gas‐exchange systems used for the measurements.
**Fig. S2** Increasing the time averaged for each data point from 1 to 10 s significantly limited the estimation noise of the leakiness.
**Fig. S3** The [CO_2_] of Li‐Cor 6400 opaque conifer chamber and Li‐Cor 6800 large leaf chamber (CO_2_S) changes with the decrease of influx [CO_2_] (CO_2_R) from 800 to 400 μmol mol^−1^.
**Fig. S4** A semilogarithmic plot of the difference between the net CO_2_ assimilation (*A*) and steady‐state net CO_2_ assimilation at 1800 μmol m^−2^ s^−1^ (*A*_f_) as a function of time.
**Fig. S5** Estimated bundle‐sheath leakiness, Δ^13^C_obs_ and *ξ* during photosynthetic induction of maize B73 calculated from tunable diode laser absorption spectroscope coupled to a gas‐exchange system (LI‐6800).
**Fig. S6** Bundle‐sheath leakiness during photosynthetic induction of maize B73 and sorghum Tx430.
**Fig. S7** Estimated *ϕ*_is_ is and *ϕ*_i_ during photosynthetic induction of sorghum and maize.
**Fig. S8** Time correction of CO_2_ assimilation and bundle‐sheath leakiness during photosynthetic induction of sorghum.
**Fig. S9** Time correction of CO_2_ assimilation and bundle‐sheath leakiness during photosynthetic induction of maize.
**Methods S1** Correction of the system delay.
**Notes S1** Performance of tunable diode laser absorption spectroscope.
**Table S1** Estimated values of leakiness and CO_2_ assimilation rate (*A*) of each individual sorghum plant.
**Table S2** Estimated values of leakiness and CO_2_ assimilation rate (*A*) of each individual maize plant.
Please note: Wiley Blackwell are not responsible for the content or functionality of any Supporting Information supplied by the authors. Any queries (other than missing material) should be directed to the *New Phytologist* Central Office.

## Data Availability

The data and code that support the findings of this study are available at doi: 10.13012/B2IDB‐1181155_V1.
