# Supplementary material for: Increased bundle‐sheath leakiness of CO2 during photosynthetic induction shows a lack of coordination between the C4 and C3 cycles
Source: New Phytol. 2022 Sep 30;236(5):1661–75. doi: 10.1111/nph.18485 (PMC9827928; doi:10.1111/nph.18485)
Supplement: Supplementary file 1 — Fig. S1 Pictures of the setup for the two gas‐exchange systems used for the measurements. Fig. S2 Increasing the time averaged for each data point from 1 to 10 s significantly limited the estimation noise of the leakiness. Fig. S3 The [CO2] of Li‐Cor 6400 opaque conifer chamber and Li‐Cor 6800 large leaf chamber (CO2S) changes with the decrease of influx [CO2] (CO2R) from 800 to 400 μmol mol−1. Fig. S4 A semilogarithmic plot of the difference between the net CO2 assimilation (A) and steady‐state net CO2 assimilation at 1800 μmol m−2 s−1 (A f) as a function of time. Fig. S5 Estimated bundle‐sheath leakiness, Δ13Cobs and ξ during photosynthetic induction of maize B73 calculated from tunable diode laser absorption spectroscope coupled to a gas‐exchange system (LI‐6800). Fig. S6 Bundle‐sheath leakiness during photosynthetic induction of maize B73 and sorghum Tx430. Fig. S7 Estimated ϕ is is and ϕ i during photosynthetic induction of sorghum and maize. Fig. S8 Time correction of CO2 assimilation and bundle‐sheath leakiness during photosynthetic induction of sorghum. Fig. S9 Time correction of CO2 assimilation and bundle‐sheath leakiness during photosynthetic induction of maize. Methods S1 Correction of the system delay. Notes S1 Performance of tunable diode laser absorption spectroscope. Table S1 Estimated values of leakiness and CO2 assimilation rate (A) of each individual sorghum plant. Table S2 Estimated values of leakiness and CO2 assimilation rate (A) of each individual maize plant. Please note: Wiley Blackwell are not responsible for the content or functionality of any Supporting Information supplied by the authors. Any queries (other than missing material) should be directed to the New Phytologist Central Office. [file NPH-236-1661-s001.pdf]

New Phytologist

***New Phytologist* Supporting Information**

Article title: Increased bundle sheath leakiness of CO_2 during photosynthetic induction shows a lack of coordination between the C_4 and C_3 cycles
Authors: Yu Wang, Samantha S. Stutz, Carl J. Bernacchi, Ryan A. Boyd, Donald R. Ort and Stephen P. Long
Article acceptance date: 25 August 2022

The following Supporting Information is available for this article:

Fig. S1 Pictures of the set up for the two gas exchange systems used for the measurements.

Fig. S2 Increasing the time averaged for each data point from 1 s to 10 s significantly limited the estimation noise of the leakiness.

Fig. S3 The [CO_2] of LICOR 6400 opaque conifer chamber and LICOR 6800 large leaf chamber (CO2S) changes with the decrease of influx [CO_2] (CO2R) from 800 µmol mol^{-1} to 400 µmol mol^{-1}.

Fig. S4 A semilogarithmic plot of the difference between the net CO_2 assimilation (*A*) and steady-state net CO_2 assimilation at 1800µmol m^{-2} s^{-1} (A_f) as a function of time.

Fig. S5 Estimated bundle-sheath leakiness, $\Delta^{13}C_{obs}$ and ξ during photosynthetic induction of maize B73 calculated from tunable diode laser absorption spectroscope (TDL) coupled to a gas exchange system (LI-6800).

Fig. S6 Bundle-sheath leakiness during photosynthetic induction of maize B73 and sorghum Tx430.

Fig. S7 Estimated ϕ_{is} and ϕ_i during photosynthetic induction of sorghum and maize.

Fig. S8 Time correction of CO_2 assimilation and bundle-sheath leakiness during photosynthetic induction of sorghum.

Fig. S9 Time correction of CO_2 assimilation and bundle-sheath leakiness during photosynthetic induction of maize.

Table S1 Estimated values of leakiness and CO2 assimilation rate (A) of each individual sorghum plant.

Table S2 Estimated values of leakiness and CO2 assimilation rate (A) of each individual maize plant.

Methods S1 Correction of the system delay

Notes S1 Performance of tunable diode laser absorption spectroscope (TDL)

Fig. S1 Pictures of the set up for the two gas exchange systems used for the measurements. Setup used with the LI-6400 for sorghum showing the LI-reference port going to the TDL on the back of the head, and the leaf chamber port at the match port (a). Setup used with the LI-6800 for maize showing the LI-reference port going to the TDL on the back of the head and the leaf chamber port on the front of the leaf chamber (b).

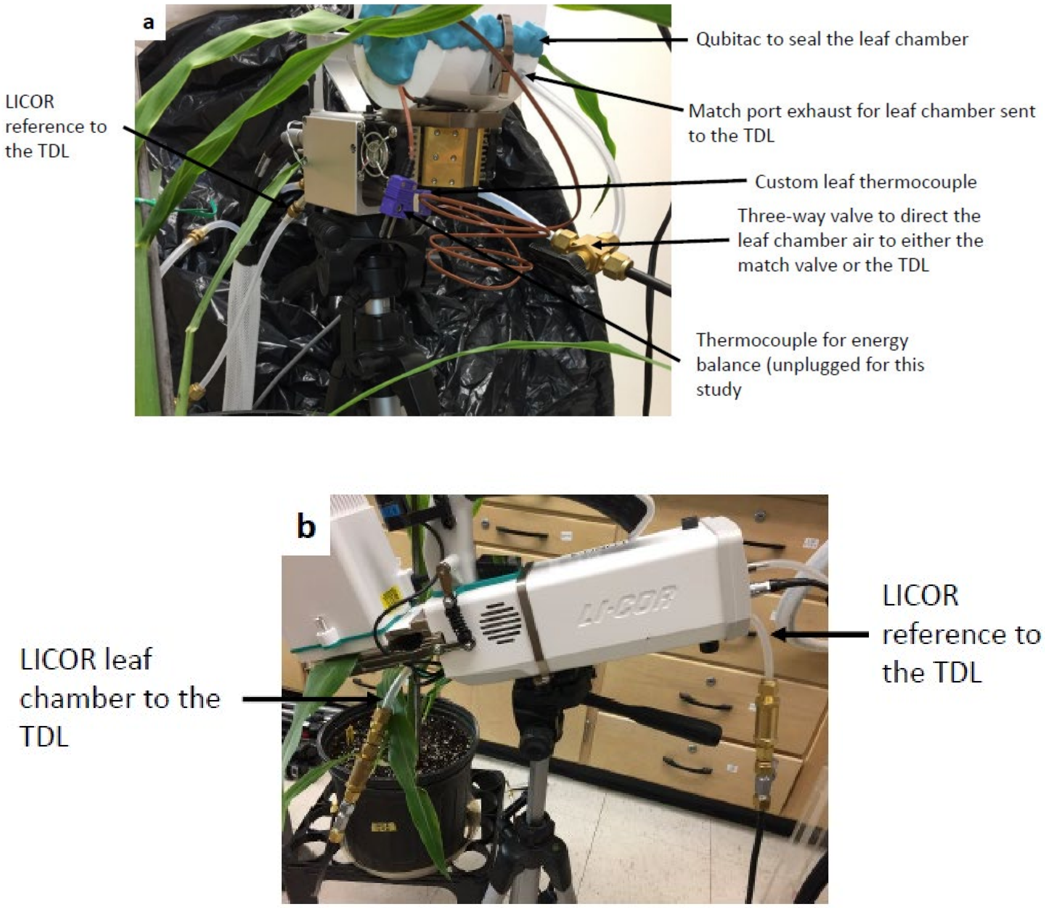

Fig. S2 Increasing the time averaged for each data point from 1 s (a) to 10 s (b) significantly limited the estimation noise of the leakiness. One measurement of sorghum is shown as an example. Time 0 s represents when the light was switched on.

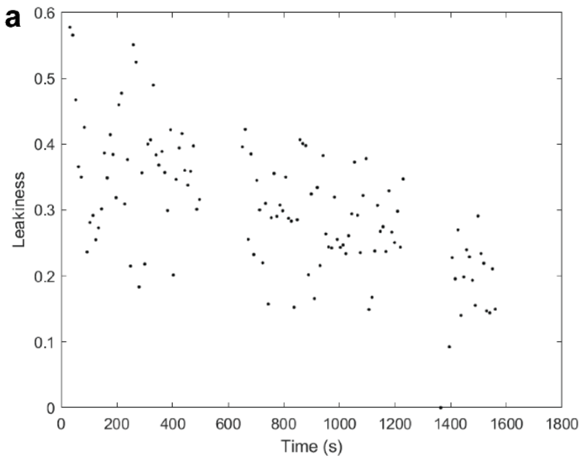

Fig. S3 The CO_2 concentration of LICOR 6400 opaque conifer chamber and LICOR 6800 large leaf chamber (CO2S) changes with the decrease of influx CO_2 concentration (CO2R) from 800 µmol mol^{-1} to 400 µmol mol^{-1}. CO2S were measured with three different flow rates, 300 µmol s^{-1}, 500 µmol s^{-1} and 700 µmol s^{-1}.

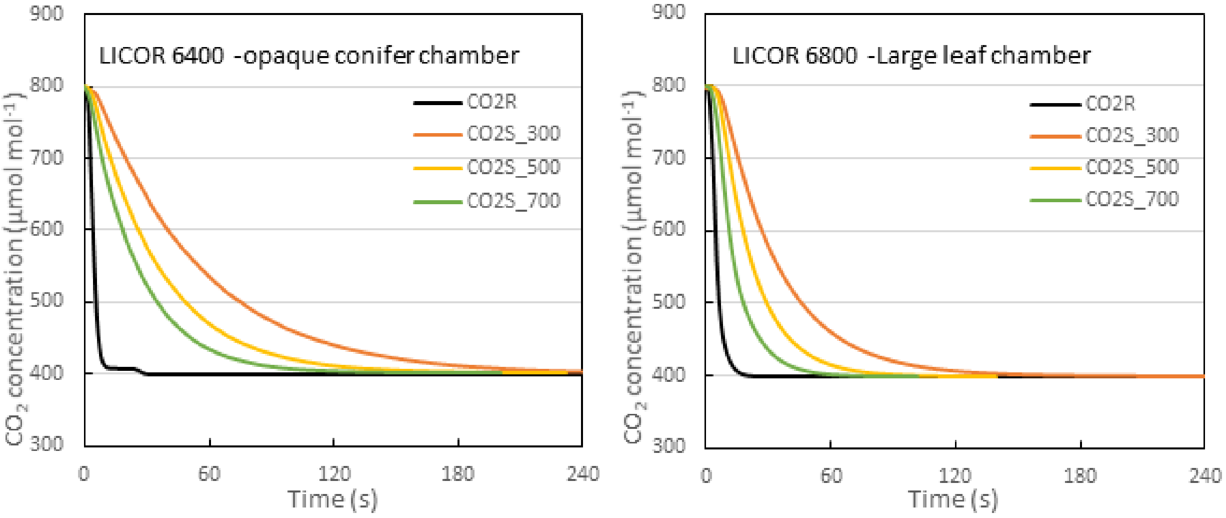

Fig. S4 A semilogarithmic plot of the difference between the net CO_2 assimilation (*A*) and steady-state net CO_2 assimilation at 1800μmol m^{-2} s^{-1} (A_f) as a function of time. Leaf gas exchange of the youngest fully expanded leaf was measured on 40 day-old sorghum Tx430 plants and maize plants. Time courses for photosynthesis were measured following a change in PPFD from 0 to 1800 μmol m^{-2} s^{-1}, time 0 s represents when the light was switched on. The time constant of Rubisco ($\tau_{Rubisco}$) was estimated from the slope of the semilogarithmic plot from 300 s to 900 s. p values were calculated using Student's t-test. Black circles represent the outliers; black lines in boxes show the medians. Upper and lower whiskers represent the maximum and minimum values, respectively.

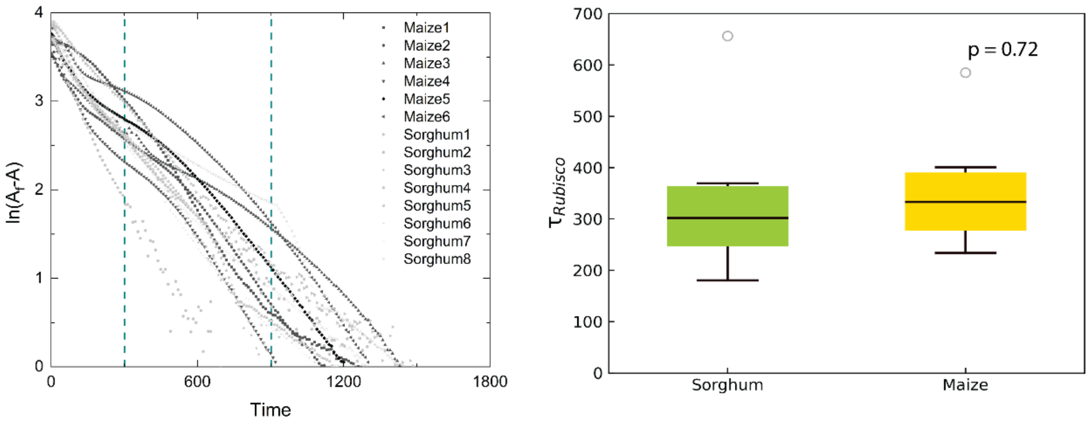

Fig. S5 Estimated bundle-sheath leakiness during photosynthetic induction of maize B73 calculated from tunable diode laser absorption spectroscope (TDL) coupled to a gas exchange system (LI-6800). a) Sample [CO_2]; b) the observed leaf photosynthetic discrimination ($\Delta^{13}C_{obs}$); c) ξ, an estimate of the uncertainty in $\Delta^{13}C_{obs}$ and ϕ calculations and d) error of $\Delta^{13}C_{obs}$ going from dark to high light (1800 μmol m^{-2} s^{-1}). Time 0 s represents when the light was switched on. Open dot represents the data point where the error of $\Delta^{13}C_{obs}$ is >50 %. Leaf gas exchange and carbon discrimination of the youngest fully expanded leaf was measured on 40 day-old maize B73 plants. The leaf was dark adapted for 30 min before the measurement. Each point of a), c) and d) is the mean (± SE) of six plants (n=6).

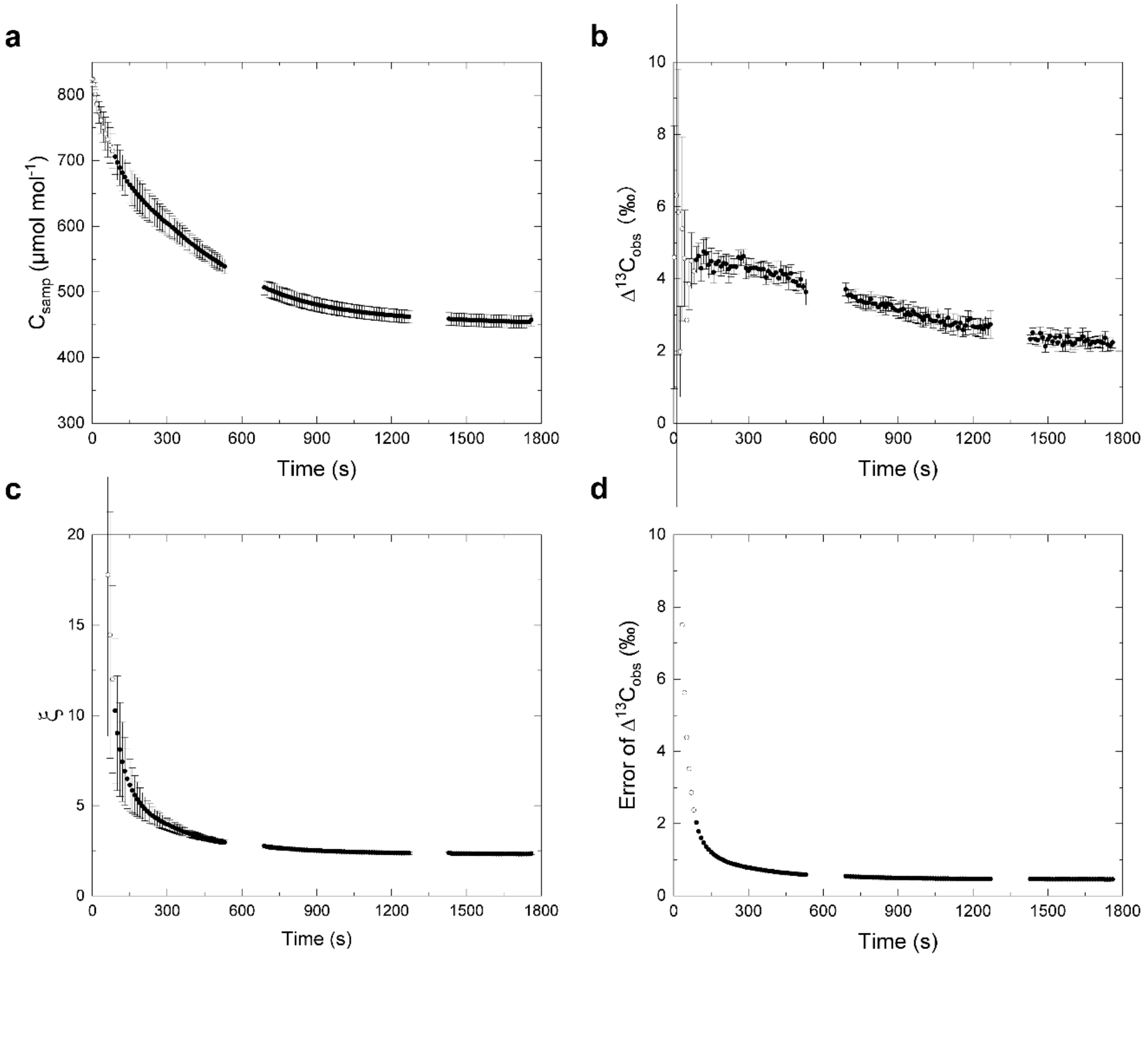

Fig. S6 Bundle-sheath leakiness during photosynthetic induction of maize B73 and sorghum Tx430. After dark adaptation, light intensity was set as 1800 µmol m-2 s-1, time 0 s represents when the light was switched on. Error bars represent ±SE.

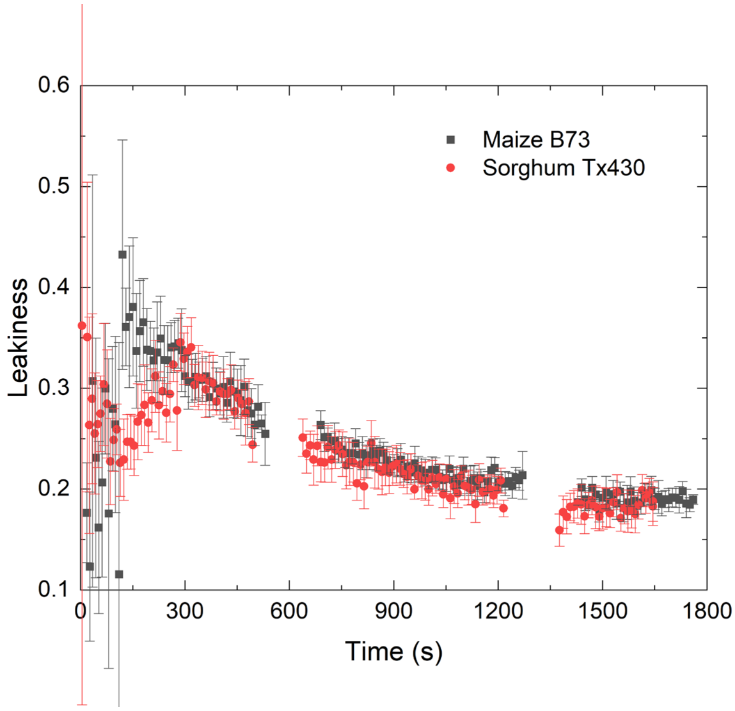

Fig. S7 Estimated ϕ_{is} and ϕ_i during photosynthetic induction of sorghum and maize. Black triangles represent the leakiness (ϕ_i) calculated from the simplified equation, assuming C_{bs} is large; Blue dots represent the leakiness (ϕ_{is}) calculated from the complete method; Green squares are the leakiness difference between the two methods at each time point. Error bars represent ±SE.

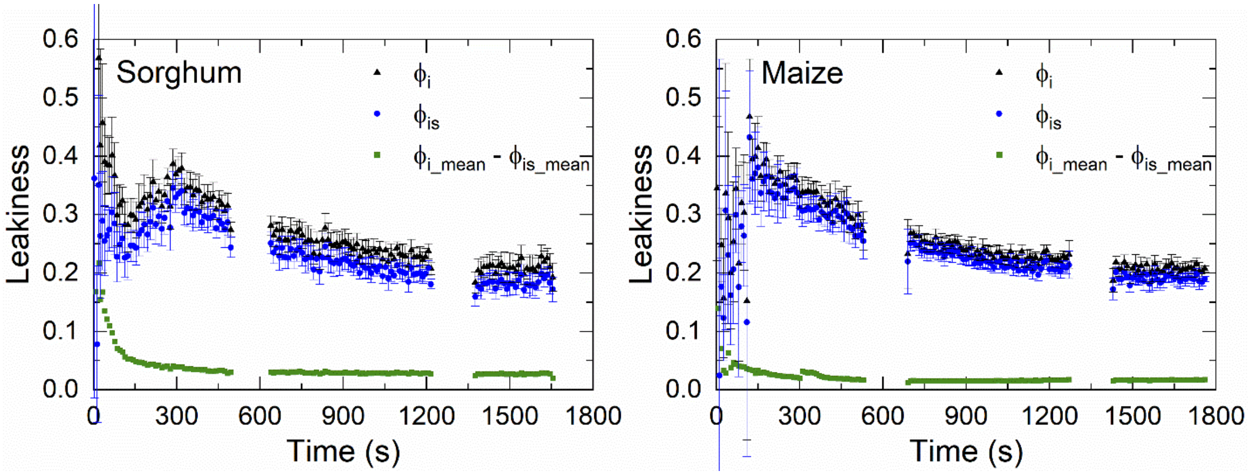

Fig. S8 Time correction of CO_2 assimilation and bundle-sheath leakiness during photosynthetic induction of sorghum measured with an LI-6400XT coupled to a TDL. a) and b) are the CO_2 assimilation rate and leakiness in the photosynthetic induction before the time correction; c) and d) are the CO_2 assimilation rate and leakiness after the time correction. Time 0 s represents when the light was switched on. Each point is the mean (± SE) of six plants. The dotted lines mark the time of highest leakiness, which is 345 s and 290 s respectively before and after correction.

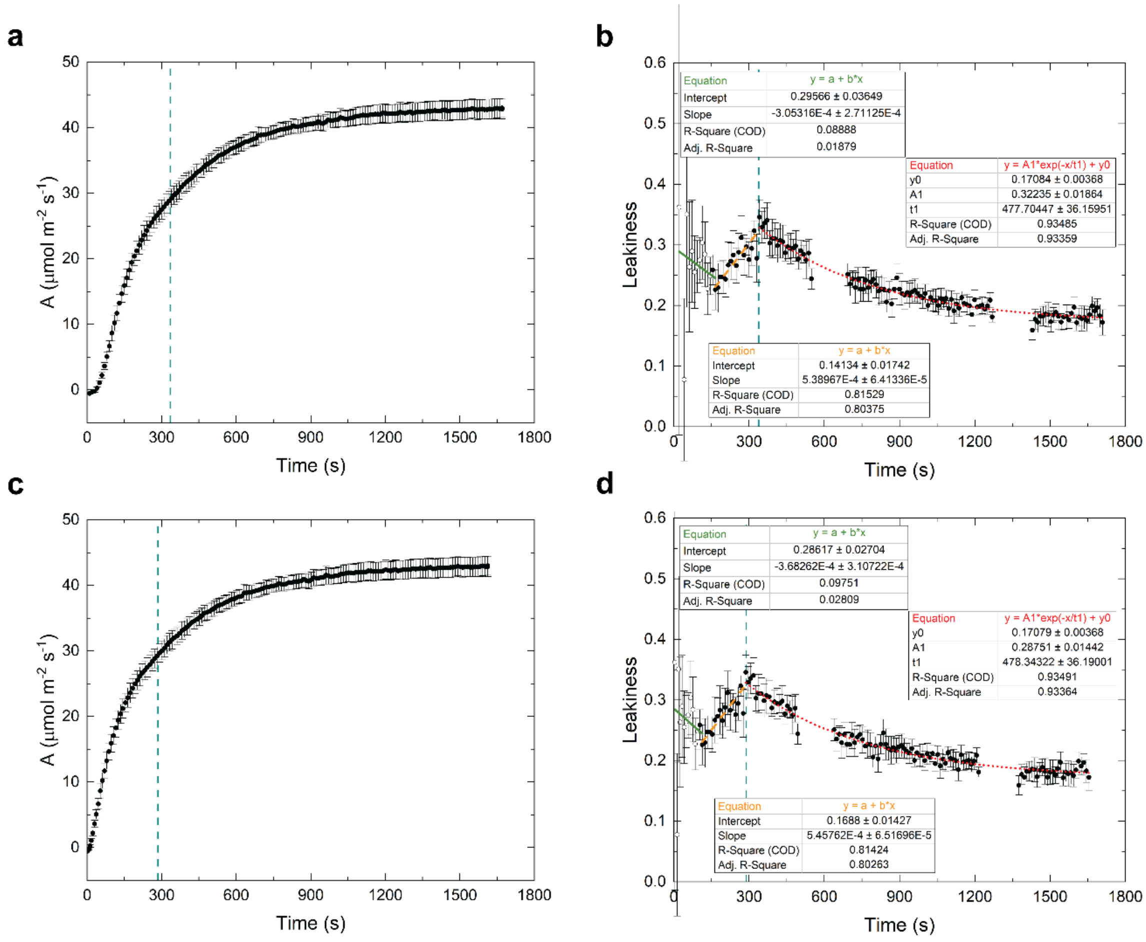

Fig. S9 Time correction of CO_2 assimilation and bundle-sheath leakiness during photosynthetic induction of maize measured with an LI-6800 coupled to a TDL. a) and b) are the CO_2 assimilation rate and leakiness in the photosynthetic induction before the time correction; c) and d) are the CO_2 assimilation rate and leakiness after the time correction. The TDL was calibrated after every 600 s of measurement. The gas from leaf chamber was not measured during the calibration and the measurement of reference gas (140 s), which occurred from ca. 530-680 s, and ca. 1280-1430 s. Time 0 s represents when the light was switched on. Each point is the mean (± SE) of six plants (n=6). The dotted lines mark the time of highest leakiness, which is 140 s and 110 s respectively before and after correction.

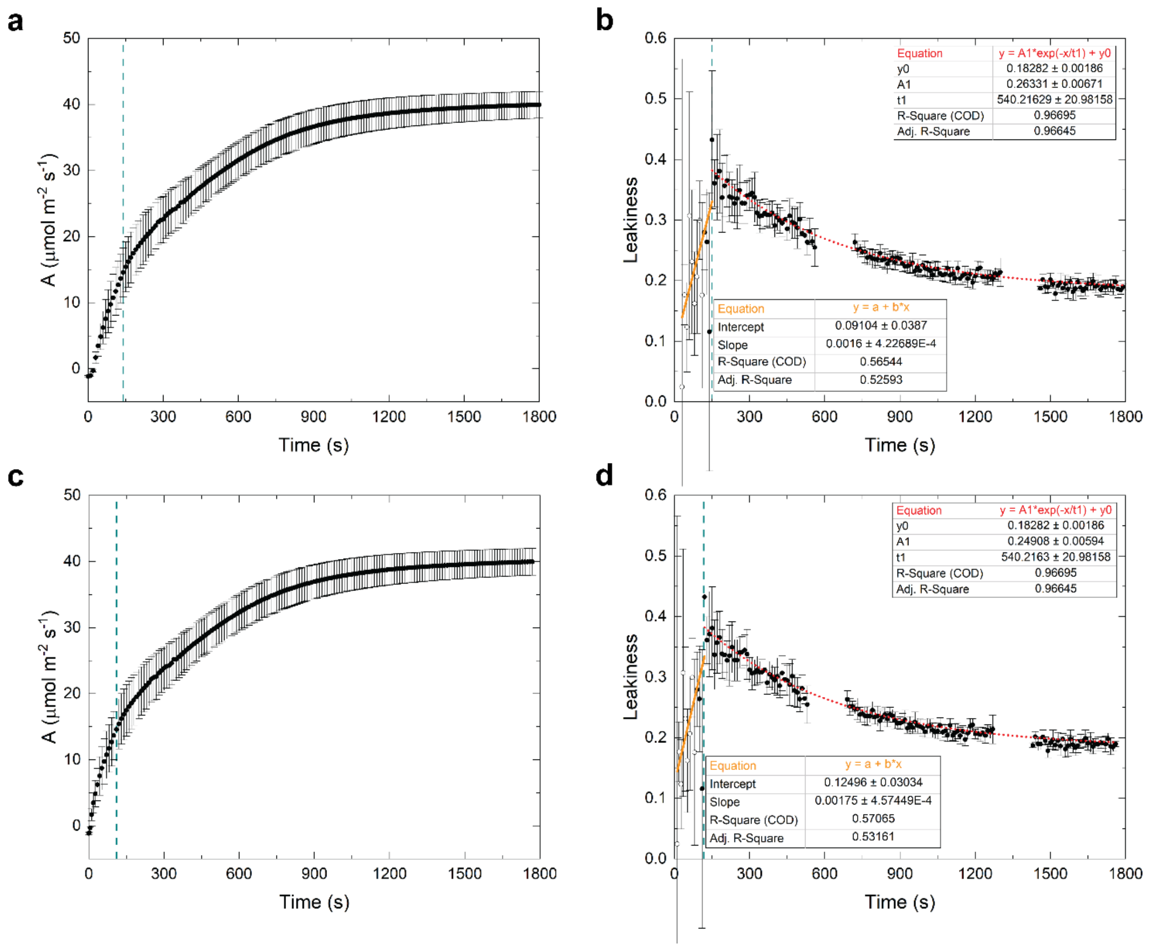

Table S1 Estimated values of leakiness and CO_2 assimilation rate (A) of each individual sorghum plant. Leak_SS: The average leakiness after 1500 s, the initial 100 s of measurement was excluded. **Leak_100-1500**: the average leakiness over the 1500 s period of induction, the initial 100 s of measurement was excluded. **Leak_100-600**: the average leakiness over the 600 s period of induction. **Leak_MT (s)**: The time at the end of the linear growth segment of leakiness. **Leak_Tao (s)**: The time constant of exponential decline segment ($\tau_{leakiness}$). **A_SS (μmol m^{-2} s^{-1})**: The average *A* after 1500 s. **A_IT50 (s):** the time that *A* reached 50% of steady state. **A_IT90 (s):** the time that *A* reached 90% of steady state *A.*

Replicate	Leak_SS	Leak_100-1500	Leak_100-600	Leak_MT	Leak_Tao	A_SS	A_IT50	A_IT90
1	0.150	0.236	0.285	266	609	47.23	237.59	754.34
2	0.170	0.238	0.285	297	436	47.16	126.26	645.47
3	0.175	0.193	0.198	287	412	38.87	187.96	633.53
4	0.135	0.198	0.272	307	197	40.29	103.29	422.47
5	0.177	0.260	0.374	287	273	40.10	158.44	667.28
6	0.252	0.244	0.266	296	152	41.88	158.58	572.13
7	0.218	0.242	0.275	307	402	40.51	158.25	664.99
8	0.164	0.286	0.353	205	603	48.75	145.19	966.76
mean	0.180	0.237	0.289	282	386	43.10	159.45	665.87
SE	0.015	0.012	0.022	14	70	1.61	16.46	63.22

Table S2 Estimated values of leakiness and CO2 assimilation rate (A) of each individual maize plant.

Replicate	Leak_SS	Leak_ 90-1500	Leak_ 90-600	Leak_MT	Leak_Tao	A_SS	A_IT50	A_IT90
1	0.190	0.270	0.356	150	553	39.62	309.79	678.96
2	0.198	0.267	0.316	140	147	38.29	127.19	737.56
3	0.159	0.251	0.271	180	600	33.58	217.96	1035.46
4	0.223	0.267	0.326	110	569	45.86	78.08	583.06
5	0.195	0.249	0.310	290	296	41.76	171.86	807.83
6	0.180	0.241	0.311	200	382	40.63	374.02	959.75
Mean	0.191	0.258	0.315	178	425	39.96	213.15	800.44
SE	0.010	0.006	0.014	32	90	2.03	55.94	85.63

Methods S1 Correction of the system delay

Time delay of gas from leaf chamber to the TDL

The time of gas from leaf chamber to the TDL was estimated by giving the leaf chamber a momentary high CO_2 and monitoring the time when the CO_2 reaches the TDL. The time it takes for the air in the leaf chamber to complete flushing the TDL optical path was 36 s and 33 s for the gas exchange systems (LI-6400 and LI-6800), respectively.

System delays caused by the volume of the leaf chamber

During the photosynthetic induction, there is a significant lag in the first minute of the CO_2 assimilation curve (Fig. S8a and Fig. S9a), which was not found in the induction curve with a standard leaf chamber (Wang et al., 2021). We assumed this lag phase was mainly due to the larger size of the leaf chamber and lower gas flow rate.

To estimate the delay due to volume and mixing in the leaf chambers, the time taken for the chamber [CO_2] (CO2S= C_{samp}) outlet air to change on switching the inlet [CO_2] (CO2R=C_{ref}) from 800 μmol mol^{-1} to 400 μmol mol^{-1} was determined for both the LI-6400XT and LI-6800 (Fig M1). A 5 cm wide paper strip was clipped into the chamber to mimic the effect of the leaf on flow and mixing. The measured data was recorded every 2 s until the chamber outlet [CO_2] was stable at 400 μmol mol^{-1}. These data were used to estimate the chamber volume ($V_{chamber}$) and time constant (τ), as defined below(https://www.licor.com/env/support/LI-6400/topics/custom-chamber.html).

Assuming the gas is well mixed in the chamber, for an open, flow-through system, the [CO_2]in the chamber *C(t)* at time t is:

$$C(t) = C_{in} - (C_{in} - C_O)e^{\frac{-t}{\tau}} \quad \text{Eqn S1}$$

where C_0 is the initial chamber [CO_2], C_{in} is the incoming [CO_2].

The time constant τ of the chamber is given by:

$$\tau = \frac{V_{chamber}}{f \cdot V_m} \quad \text{Eqn S2}$$

where V_m is the molar volume of air, which was assumed to approximate to an ideal gas an ideal gas at standard atmospheric pressure and 28°C, it is set as 24.7 l mol^{-1}. f is the air flow rate and $V_{chamber}$ is the chamber volume.

The time constant of the CO2S response (τ) was estimated using eqn. S2 (Table SI-1). These time constants of the chamber CO_2 response were used to calculate the chamber volume and the system delay time (Table M1, Fig. M2, Eqn. S4, 5). The estimated chamber volume of the opaque conifer chamber is 0.371 L and volume of the large leaf chamber is 0.212 L with the flow rate 300 µmol s^{-1}. The estimated values are almost the same regardless of flow rate, because the maximum difference is about 8% (Table SI-1), indicating the estimated chamber volume is reliable and the gas in the leaf chamber is well mixed.

An ordinary differential equation model was used to estimate the system delay during photosynthetic induction measurement.

$$\frac{dC}{dt} = \frac{A_{leaf}(C) - A'_{leaf}}{V_{chamber}} V_m \qquad \text{Eqn S3}$$

where A_{leaf} is the leaf photosynthesis rate estimated by the gas exchange system: A'_{leaf} is the actual photosynthesis rate, here we set it as:

$$A'_{leaf} = A_f(1 - e^{-\frac{t}{\tau_A}}) \qquad \text{Eqn S4}$$

where S_{leaf} is the leaf area, A_f is the steady state photosynthesis rate at high light. τ_A is the time constant of the induction of photosynthesis. A_f and τ_A were set as 40 µmol m^{-2} s^{-1} and 300 s according to the gas exchange measurements

Compared with the actual CO_2 assimilation rate, the increase of measured CO_2 assimilation was slower, and the difference is more significant in the first few minutes of the induction (Fig. M2a). The delay time increased from 0 to 54 s within the first 200 s, and then stabilized around 54 s for the opaque conifer chamber with LI-6400XT (Fig. M2b); and the maximal delay time for the large leaf and needle (6800-13) chamber with LI-6800 was 30s. Compared with the original results, there is no lag phase in the first minute of the corrected photosynthetic induction curve (Fig. S8a, c; Fig. S9a, c). This validates our hypothesis that the

lag in the photosynthetic induction curve is caused by the delay of the experimental system. Meanwhile, it also indicated that the system delay can be effectively corrected.

After the correction, the highest leakiness was found at about 286 s instead of 340s for sorghum. In maize, the time of highest leakiness was 140 s and 110 s respectively before and after correction (Fig S8b, d; Fig S9b, d).

Table M1 Time constant of chamber CO_2 response and estimated chamber volume

Flow rate	Time constant of CO2S response (τ)	Estimated Camber volume ($V_{chamber}$)
**LI-6400XT and the opaque conifer chamber**		
**300 μmol s^{-1}**	50.0 s	0.369L
**500 μmol s^{-1}**	32.3 s	0.397 L
**700 μmol s^{-1}**	23.3 s	0.400 L
**LI 6800 and the large leaf and needle (6800-13) chamber**		
**300 μmol s^{-1}**	28.6 s	0.211 L
**500 μmol s^{-1}**	16.6 s	0.204 L
**700 μmol s^{-1}**	11.4 s	0.196 L

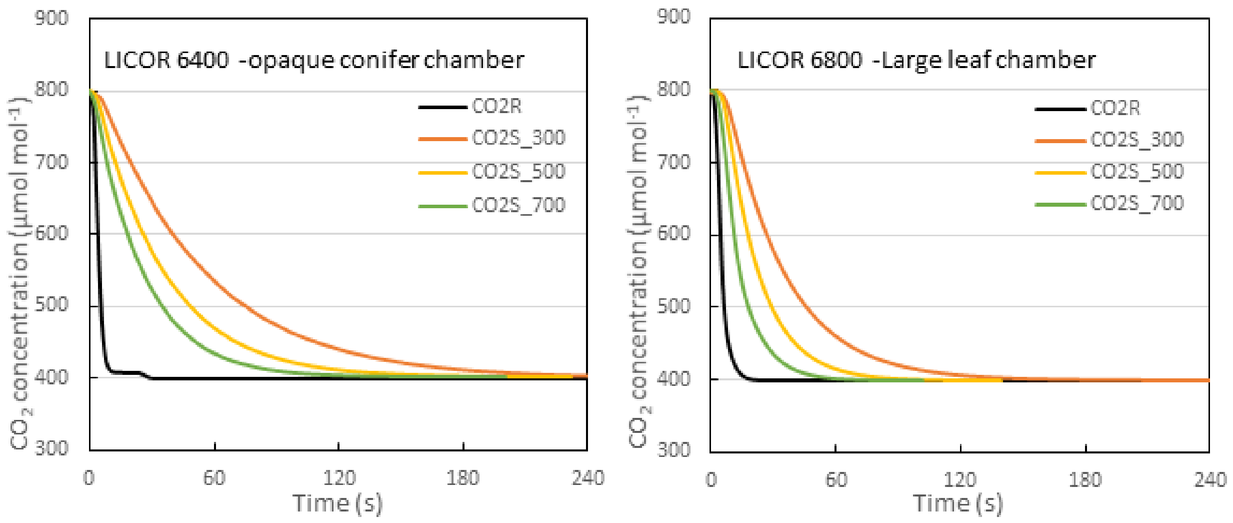

Figure M1 the CO_2 concentration of LICOR 6400 opaque conifer chamber and LICOR 6800 large leaf chamber (CO2S=C_{samp}) changes with the decrease of influx CO_2 concentration (CO2R= C_{ref}) from 800 µmol mol^{-1} to 400 µmol mol^{-1}. CO2S were measured with three different flow rates, 300 µmol s^{-1}, 500 µmol s^{-1} and 700 µmol s^{-1}.

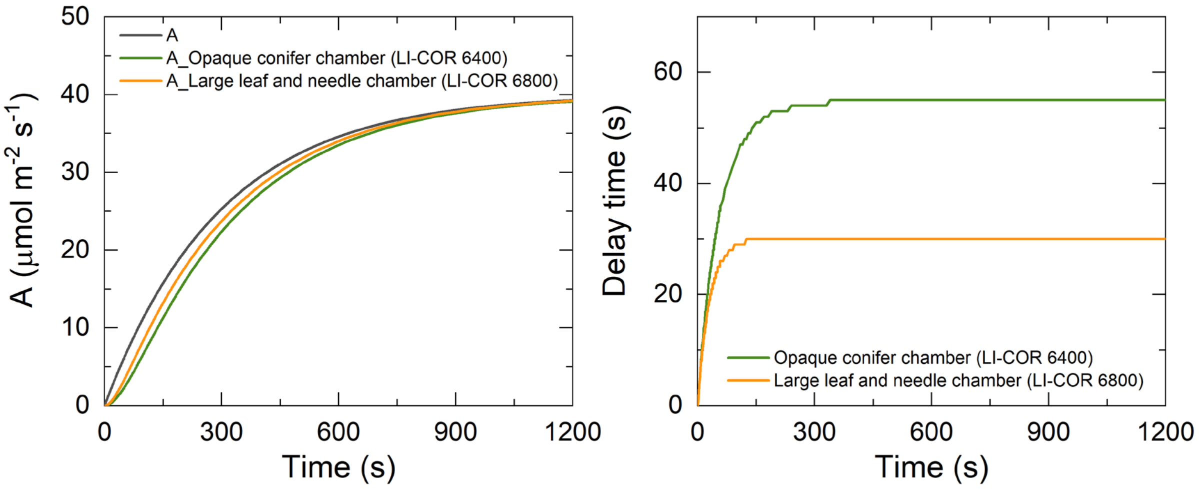

Figure M2 The actual CO_2 assimilation rate and the estimated CO_2 assimilation rate from gas exchange measurement with the opaque conifer chamber (LI-6400XT) and the large leaf and needle chamber (LI-6800). Flow rate was set as 300 µmol s^{-1}.

Notes S1 Performance of tunable diode laser absorption spectroscope (TDL)

1. Allan deviations and drift of the TDL

To estimate Allan deviation, we measured Airgas standard gas with a [$^{12}CO_2$] of 494 µmol mol^{-1}, [$^{13}CO_2$] of 5.3 µmol mol^{-1} and δ^{13}C of -40.4‰ at 21% [O_2] (Airgas, Plumsteadville, PA, USA). for at least 8 hours. Allan deviations of the raw data of the 8-hour measurement were shown in Fig. N1. The Allan variance (avar) were estimated using a MATLAB function (allanvar), the Allan deviation(adev) is the square root of the Allan variance (adev = sqrt(avar)).

As shown in Fig. N1, the standard deviation of noise in $^{12}CO_2$ of a single point is about 0.2 µmol mol^{-1} (0.4 ‰) for each measurement. The minimums on an Allan deviation graph are ca. 0.015 µmol mol^{-1} (0.04 ‰), and the optimal sample times (τ) are ca. 20-30 s. Because of the low frequency noise (drift), the Allan deviations increased to 0.06 µmol mol^{-1} (0.09 ‰) at sample time of 740 s (τ=740s), which is the time of a complete cycle of calibration and measurement. The standard deviation of noise in $^{13}CO_2$ of a single point is about 0.008 µmol mol^{-1} (1.5 ‰) for each measurement. The minimums on an Allan deviation graph are ca. 0.0004 µmol mol^{-1} (0.08 ‰), and the optimal sample times (τ) are ca. 50-100 s. The Allan deviation was less than 0.0006 µmol mol^{-1} (0.11 ‰) at sample time of 740 s (τ=740s).

We used 10 s as the sample time in our data processing. With this setting, the Allan deviation of $^{12}CO_2$ is only ca. 0.02 µmol mol^{-1} (0.04 ‰); The Allan deviation of $^{13}CO_2$ is ca.0.0008 µmol mol^{-1} (0.15 ‰). Although measuring over 740s increases the Allan deviation, the error is only 0.09‰ and 0.11‰ for $^{12}CO_2$ and $^{13}CO_2$, respectively.

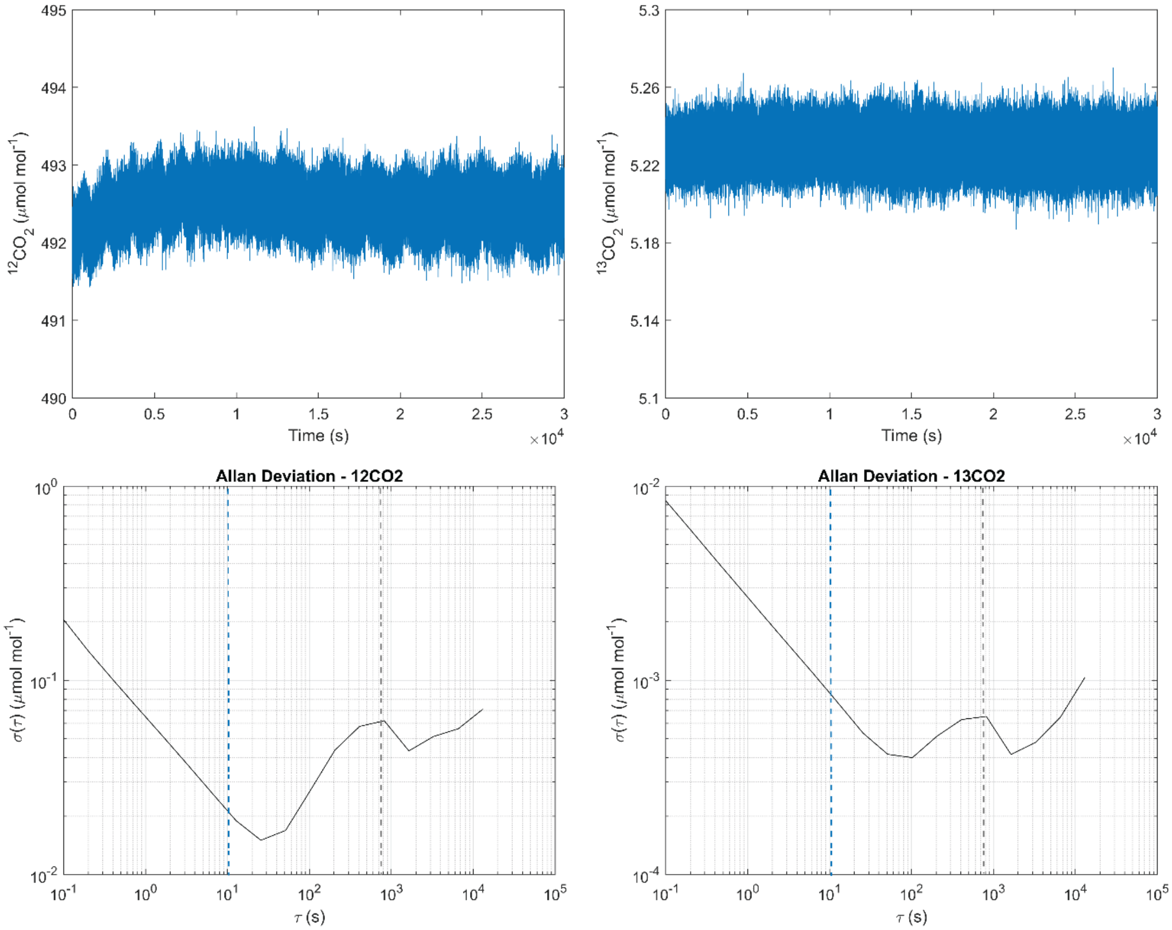

Figure N1 Measured $^{12}CO_2$ and $^{13}CO_2$ concentration and Allan deviations of the CO_2 sensors. The blue and gray dash lines show the time of 10s and 740s, respectively.

2. Precision of the TDL

Instrument precision was estimated according to Ubierna *et al.*, 2018, where we calculated the mean $\delta^{13}C$ over the last 10 s of the measurement cycle for the NOAA calibration tank in the calibration series for the measurements from each day. This accounted for between 15 and 34 points. We then calculated standard deviation from these measurements. The precision for the measurements of sorghum was 0.24‰, the instrument precision for the measurements of maize was 0.14‰. It is likely that the difference in precision between the measurements was the result of a major retuning of the laser that occurred in February 2020 after the sorghum measurements and before the maize measurements. Because of the difference in precision, we

chose to use the precision for sorghum (0.24‰) and the precision for maize (0.14‰) with the respective measurements.

3. Error of $\Delta^{13}C_{obs}$

The error associated with each measurement was calculated according to Ubierna *et al.,* (2018) as:

$$error\ (‰) = \sqrt{2} \cdot \xi \cdot X \qquad \text{Eqn SN 1}$$

where, ξ is the uncertainty in the measurement of $\Delta^{13}C_{obs}$ calculated according to Evans *et al.*, (1986), as:

$$\frac{CO_{2\,ref}}{CO_{2\,ref} - CO_{2\,samp}} \qquad \text{Eqn SN 2}$$

where, CO_{2ref} is the $[CO_2]$ of the air entering the leaf chamber and CO_{2samp} is the $[CO_2]$ of the air exiting the leaf chamber. *X* is the precision of the tunable diode laser (TDL). We used the precision estimated above for sorghum and maize, respectively. The error associated with the measurement shows us the range that $\Delta^{13}C_{obs}$ can very. For instance, if $\Delta^{13}C_{obs}$ is 3.7‰ and the error is 1.5‰ the associated error would account for 42% of $\Delta^{13}C_{obs}$. We observed that the error was over 50% in the first 144 s and 110 s for sorghum and maize, respectively. The open circles in Fig. 1 and S5 represent these data. We also chose to limit the interpretation of our measurements during this time since our error is large. The error associated with the measurements declines quickly and by 300 s makes up only 28% of the measurement for sorghum and 18% of the measurement for maize. Table N1 and N2 show the ξ, $\Delta^{13}C_{obs}$, associated error and the percentage of the error in the measurement.

Table N1. Average of photosynthetic discrimination ($\Delta^{13}C_{obs}$), ξ, error associated with the percent of $\Delta^{13}C_{obs}$ that is accounted by the error in sorghum. Sorghum plants were measured with the LI-6400 using the opaque conifer chamber. Yellow highlighting shows the measured values that were excluded for further analysis.

Time (s)	$\Delta^{13}C_{obs}$ (‰)	ξ	Error	% Error
**1.0**	-21.9	-271.8	-90.9	426.9
**3.8**	605.0	-417.8	-139.7	-23.1

**8.0**	77.2	-288.7	-96.5	-125
**13.0**	-13.1	-123.3	-41.2	315.2
**18.8**	9.7	70.8	23.67	245.0
**25.1**	5.1	33.5	11.2	219.7
**33.0**	6.1	22.1	7.4	120.5
**40.8**	5.3	16.1	5.4	101.1
**49.1**	5.3	12.5	4.2	79.5
**57.3**	5.3	10.3	3.5	64.8
**66.8**	5.1	8.7	2.9	57.4
**76.0**	4.3	7.6	2.5	59.1
**85.3**	3.9	6.8	2.3	57.3
**94.9**	3.9	6.1	2.0	52.6
**104.0**	4.0	5.6	1.9	47.0
**113.4**	3.6	5.2	1.8	49.1
**123.9**	3.6	4.9	1.6	45.4
**134.0**	3.7	4.7	1.6	42.2
**143.5**	3.7	4.5	1.5	39.7
**153.9**	3.6	4.3	1.4	39.4
**164.3**	3.9	4.1	1.4	34.9
**174.5**	4.0	4.0	1.3	33.2
**183.9**	4.0	4.0	1.3	32.3
**194.5**	3.9	3.7	1.2	32.4
**204.6**	4.2	3.6	1.2	28.9
**214.9**	4.6	3.6	1.2	26.0
**225.3**	4.2	3.5	1.2	27.8
**235.5**	4.3	3.4	1.1	26.3
**245.9**	4.0	3.4	1.1	28.3
**256.4**	4.3	3.3	1.1	25.8
**266.6**	4.6	3.2	1.9	23.7
**276.9**	3.9	3.2	1.1	27.3
**286.5**	4.8	3.1	1.1	21.8
**296.5**	4.7	3.1	1.0	22.1
**306.8**	4.8	3.1	1.0	21.4
**317.4**	4.7	3.0	1.0	21.5
**327.5**	4.4	3.0	1.0	22.6
**337.8**	4.3	3.0	1.0	22.9
**349.4**	4.4	2.9	1.0	22.2
**359.6**	4.1	2.9	1.0	23.1
**369.8**	4.2	2.8	0.9	22.5
**380.5**	4.2	2.8	0.9	22.2
**390.6**	4.0	2.8	0.9	23.0
**400.8**	4.2	2.7	0.9	21.8
**411.6**	4.1	2.6	1.3	31.1
**421.7**	4.1	2.6	1.2	29.8

**432.3**	4.2	2.6	0.9	20.7
**442.7**	3.9	2.7	0.9	23.1
**452.8**	4.1	2.7	0.9	22.0
**463.2**	4.1	2.7	0.9	22.0
**473.5**	3.8	2.6	0.9	23.1
**483.8**	3.9	2.6	0.9	22.2
**494.3**	3.1	2.6	0.9	28.5
**638.8**	3.3	2.4	0.8	24.7
**648.9**	3.1	2.9	0.8	25.9
**659.4**	3.2	2.8	0.8	25.2
**669.6**	3.0	2.4	0.8	26.6
**679.8**	3.3	2.4	0.8	24.4
**690.5**	3.0	2.4	0.8	26.6
**700.8**	2.9	2.4	0.8	27.7
**710.9**	3.1	2.4	0.8	25.3
**721.5**	2.9	2.4	0.8	27.0
**732.0**	3.1	2.4	0.8	25.5
**742.1**	3.2	2.4	0.8	24.4
**752.4**	3.0	2.4	0.8	26.2
**762.9**	2.8	2.4	0.8	27.8
**773.0**	2.9	2.3	0.8	27.2
**783.4**	2.8	2.3	0.8	27.8
**793.8**	2.5	2.3	0.8	31.3
**804.3**	2.8	2.3	0.8	27.8
**814.4**	2.4	2.3	0.8	31.9
**824.8**	2.8	2.3	0.8	27.6
**835.3**	3.3	2.3	0.8	23.8
**845.4**	2.9	2.3	0.8	26.7
**855.8**	2.7	2.3	0.8	28.8
**866.3**	2.8	2.3	0.8	27.6
**876.4**	2.8	2.3	0.8	27.7
**886.6**	2.6	2.3	0.8	29.3
**897.1**	2.7	2.3	0.8	28.2
**907.3**	2.8	2.3	0.8	27.2
**917.8**	2.7	2.3	0.8	28.1
**928.1**	2.6	2.3	0.8	29.4
**938.3**	2.7	2.3	0.8	29.0
**948.8**	2.8	2.3	0.8	27.4
**959.1**	2.4	2.3	0.8	32.3
**969.4**	2.7	2.3	0.8	28.8
**980.1**	2.5	2.3	0.8	30.8
**990.1**	2.5	2.3	0.8	33.8
**1000.3**	2.3	2.3	0.8	31.5
**1010.8**	2.5	2.3	0.8	31.5

**1021.1**	2.4	2.3	0.8	32.8
**1031.3**	2.5	2.3	0.8	31.4
**1041.8**	2.2	2.3	0.8	35.3
**1052.1**	2.4	2.3	0.8	32.2
**1062.4**	2.1	2.3	0.8	36.2
**1072.8**	2.3	2.3	0.8	33.5
**1083.3**	2.1	2.3	0.8	36.0
**1093.5**	2.5	2.3	0.8	31.2
**1103.8**	2.3	2.3	0.8	33.1
**1114.1**	2.3	2.3	0.8	33.7
**1124.4**	2.3	2.3	0.8	34.0
**1134.6**	2.0	2.3	0.8	39.0
**1145.1**	2.5	2.3	0.8	31.4
**1155.4**	2.3	2.3	0.8	34.2
**1165.8**	2.2	2.3	0.8	35.0
**1176.0**	2.3	2.3	0.8	34.0
**1186.3**	2.1	2.3	0.8	36.3
**1196.5**	2.2	2.3	0.8	35.2
**1207.1**	2.3	2.3	0.8	32.7
**1215.2**	1.7	2.4	0.8	46.5
**1375.1**	1.9	2.6	0.9	47.4
**1386.4**	1.8	2.3	0.8	41.5
**1396.9**	1.8	2.3	0.8	43.3
**1407.1**	1.9	2.3	0.8	39.7
**1417.6**	2.0	2.3	0.8	39.0
**1427.9**	2.0	2.3	0.8	38.8
**1438.1**	2.0	2.3	0.8	38.9
**1448.5**	1.8	2.3	0.8	42.7
**1459.1**	1.9	2.3	0.8	39.1
**1469.4**	2.1	2.3	0.8	36.9
**1479.5**	2.0	2.3	0.8	38.9
**1490.1**	1.7	2.3	0.8	43.8
**1500.3**	2.0	2.3	0.8	38.6
**1510.8**	1.9	2.3	0.8	39.1
**1521.1**	1.8	2.3	0.8	41.9
**1531.3**	2.0	2.3	0.8	38.3
**1541.6**	2.2	2.3	0.8	34.
**1552.0**	1.7	2.3	0.8	45.2
**1562.1**	1.9	2.3	0.8	40.8
**1572.5**	1.9	2.3	0.8	40.3
**1582.8**	1.9	2.3	0.8	39.6
**1593.0**	1.8	2.3	0.8	41.7
**1603.3**	1.9	2.3	0.8	38.8
**1613.6**	2.3	2.3	0.8	33.3

**1624.0**	2.1	2.3	0.8	36.1
**1634.3**	2.2	2.3	0.8	35.0
**1644.5**	2.0	2.3	0.8	38.7
**1654.9**	1.6	2.3	0.8	46.4

Table N2. Photosynthetic discrimination ($\Delta^{13}C_{obs}$), ξ, error associated with the measurement and the percent of $\Delta^{13}C_{obs}$ that is accounted by the error in maize. Maize plants were measured with the LI-6800 using the large leaf and needle chamber. Yellow highlighting shows the measured values that were excluded for further analysis.

Time (s)	$\Delta^{13}C_{obs}$ (‰)	ξ	Error	% Error
**2**	4.6	-95.2	-19.0	-413.5
**6**	-200.4	-7495.1	-1500.0	748.6
**11**	6.3	-263.0	-52.6	-832.9
**18**	5.9	121.0	24.2	413.7
**26**	2.0	55.9	11.2	561.9
**34**	5.4	37.9	7.6	140.7
**43**	4.6	28.5	5.7	124.6
**52**	2.9	22.2	4.4	155.8
**62**	3.9	17.8	3.6	92.2
**71**	4.5	14.5	2.9	64.2
**81**	4.2	12.0	2.4	57.0
**91**	4.5	10.3	2.1	45.3
**100**	4.6	9.0	1.8	39.0
**110**	4.3	8.1	1.6	37.8
**120**	4.8	7.4	1.5	31.2
**130**	4.7	6.9	1.4	29.3
**140**	4.4	6.5	1.3	29.6
**150**	4.5	6.1	1.2	27.3
**160**	4.2	5.8	1.2	27.9
**170**	4.4	5.6	1.1	25.3
**180**	4.5	5.4	1.1	23.8
**190**	4.4	5.2	1.0	23.7
**200**	4.4	5.0	1.0	22.5
**210**	4.4	4.8	1.0	21.8
**220**	4.3	4.7	0.9	22.0
**230**	4.4	4.6	0.9	21.0
**240**	4.4	4.5	0.9	20.5
**250**	4.3	4.4	0.9	20.1
**260**	4.6	4.3	0.9	18.6
**270**	4.6	4.2	0.8	18.3
**280**	4.6	4.1	0.8	17.7
**290**	4.3	4.0	0.8	18.8
**300**	4.2	4.0	0.8	18.8

**310**	4.3	3.9	0.8	18.1
**320**	4.3	3.8	0.8	17.8
**330**	4.3	3.8	0.8	17.5
**340**	4.3	3.7	0.7	17.4
**350**	4.3	3.7	0.7	17.2
**360**	4.3	3.6	0.7	16.9
**370**	4.0	3.5	0.7	17.5
**380**	4.2	3.5	0.7	16.6
**390**	4.1	3.4	0.7	16.7
**400**	4.1	3.4	0.7	16.6
**410**	4.2	3.4	0.7	16.1
**420**	4.0	3.3	0.7	16.5
**430**	4.2	3.3	0.7	15.5
**440**	4.1	3.2	0.6	15.7
**450**	4.1	3.2	0.6	15.6
**460**	4.0	3.2	0.6	15.8
**470**	4.2	3.1	0.6	15.1
**480**	3.9	3.1	0.6	15.8
**490**	3.9	3.1	0.6	15.7
**500**	3.8	3.1	0.6	16.0
**510**	4.0	3.0	0.6	15.2
**520**	3.8	3.0	0.6	15.9
**530**	3.6	3.0	0.6	16.3
**690**	3.6	2.7	0.5	15.2
**700**	3.6	2.7	0.5	15.3
**710**	3.6	2.7	0.5	15.2
**720**	3.5	2.7	0.5	15.3
**730**	3.5	2.7	0.5	15.4
**740**	3.4	2.7	0.5	15.7
**750**	3.4	2.7	0.5	15.5
**760**	3.3	2.6	0.5	15.9
**770**	3.3	2.6	0.5	15.9
**780**	3.4	2.6	0.5	15.3
**790**	3.4	2.6	0.5	15.4
**800**	3.3	2.6	0.5	15.7
**810**	3.3	2.6	0.5	15.7
**820**	3.2	2.6	0.5	16.0
**830**	3.3	2.6	0.5	15.5
**840**	3.3	2.6	0.5	15.6
**850**	3.3	2.5	0.5	15.6
**860**	3.3	2.5	0.5	15.3
**870**	3.3	2.5	0.5	15.6
**880**	3.2	2.5	0.5	15.9
**890**	3.1	2.5	0.5	16.2

**900**	3.2	2.5	0.5	15.9
**910**	3.1	2.5	0.5	16.1
**920**	3.2	2.5	0.5	15.8
**930**	3.0	2.5	0.5	16.6
**940**	3.1	2.5	0.5	16.3
**950**	3.1	2.5	0.5	16.1
**960**	3.1	2.5	0.5	16.2
**970**	3.0	2.5	0.5	16.4
**980**	2.9	2.5	0.5	16.9
**990**	3.0	2.5	0.5	16.6
**1000**	2.9	2.5	0.5	17.0
**1010**	3.0	2.4	0.5	16.6
**1020**	2.9	2.4	0.5	16.7
**1030**	2.9	2.4	0.5	16.7
**1040**	2.8	2.4	0.5	17.4
**1050**	2.9	2.4	0.5	16.8
**1060**	2.9	2.4	0.5	16.6
**1070**	2.8	2.4	0.5	17.4
**1080**	2.8	2.4	0.5	17.4
**1090**	2.7	2.4	0.5	17.7
**1100**	2.9	2.4	0.5	16.8
**1110**	2.8	2.4	0.5	17.5
**1120**	2.7	2.4	0.5	17.7
**1130**	2.8	2.4	0.5	17.1
**1140**	2.7	2.4	0.5	18.0
**1150**	2.7	2.4	0.5	17.5
**1160**	2.6	2.4	0.5	18.8
**1170**	2.8	2.4	0.5	17.3
**1180**	2.9	2.4	0.5	16.6
**1190**	2.8	2.4	0.5	16.9
**1200**	2.7	2.4	0.5	17.8
**1210**	2.7	2.4	0.5	17.4
**1220**	2.6	2.4	0.5	18.1
**1230**	2.7	2.4	0.5	17.9
**1240**	2.6	2.4	0.5	18.4
**1250**	2.7	2.4	0.5	17.6
**1260**	2.7	2.4	0.5	17.3
**1270**	2.7	2.4	0.5	17.5
**1430**	2.4	2.4	0.5	19.5
**1440**	2.5	2.4	0.5	18.8
**1450**	2.3	2.4	0.5	20.4
**1460**	2.3	2.3	0.5	20.0
**1470**	2.4	2.3	0.5	19.2
**1480**	2.4	2.3	0.5	19.9

**1490**	2.1	2.3	0.5	21.9
**1500**	2.4	2.3	0.5	19.6
**1510**	2.3	2.3	0.5	20.1
**1520**	2.4	2.3	0.5	19.8
**1530**	2.4	2.3	0.5	19.6
**1540**	2.2	2.3	0.5	21.4
**1550**	2.4	2.3	0.5	19.5
**1560**	2.2	2.3	0.5	21.4
**1570**	2.3	2.3	0.5	20.6
**1580**	2.3	2.3	0.5	20.0
**1590**	2.3	2.3	0.5	20.7
**1600**	2.2	2.3	0.5	21.5
**1610**	2.2	2.3	0.5	21.1
**1620**	2.3	2.3	0.5	20.1
**1630**	2.4	2.3	0.5	19.3
**1640**	2.3	2.3	0.5	20.0
**1650**	2.3	2.3	0.5	20.0
**1660**	2.3	2.3	0.5	20.6
**1670**	2.1	2.3	0.5	21.7
**1680**	2.3	2.3	0.5	20.7
**1690**	2.2	2.3	0.5	20.7
**1700**	2.3	2.3	0.5	20.3
**1710**	2.3	2.3	0.5	20.4
**1720**	2.3	2.3	0.5	20.6
**1730**	2.3	2.3	0.5	20.1
**1740**	2.1	2.3	0.5	21.8
**1750**	2.1	2.3	0.5	21.6
**1760**	2.2	2.3	0.5	20.7

References

Evans JR, Sharkey TD, Berry JA, GD Farquhar. 1986. Carbon isotope discrimination measured concurrently with gas exchange to investigate CO_2 diffusion in leaves of higher plants. *Australian Journal of Plant Physiology* **13**: 281-292.

Ubierna N, Holloway-Phillips MM, Farquhar GD. 2018. Using stable carbon isotopes to study C_3 and C_4 photosynthesis: models and calculations. In: Covshoff S (ed) *Photosynthesis. Methods in molecular biology*, vol 1770 Humana Press, New York, pp 155-196.

Wang Y, Chan KX, Long SP. 2021. Toward a dynamic photosynthesis model to guide yield improvement in C4 crops. *The Plant Journal* **107**(2), 343-359.
